# Inf–Sup Stable Space–Time Discretization of the Wave Equation Based on a First-Order-In-Time Variational Formulation

**DOI:** 10.1007/s10915-026-03293-w

**Published:** 2026-05-04

**Authors:** Matteo Ferrari, Ilaria Perugia, Enrico Zampa

**Affiliations:** Faculty of Mathematics, University of Vienna, Oskar-Morgenstern-Platz 1, 1090 Vienna, Austria

**Keywords:** Wave equation, Space–time method, First-order-in-time formulation, Inf–sup stability, 65M60, 65N12

## Abstract

In this paper, we present a conforming space–time discretization of the wave equation based on a first-order-in-time variational formulation. Our method extends the scheme of French and Peterson (1996), incorporating exponential weights in time, which yield an inf–sup stability condition for arbitrary choices of discrete subspaces, including spline spaces, without restrictions on the mesh size or time step. Moreover, using elliptic projections, we derive optimal convergence rates in both the energy and $L^2$ norms for sufficiently smooth solutions and for any choice of space–time tensor product subspaces satisfying standard approximation assumptions. Numerical examples are provided to support the theoretical findings.

## Introduction

The need for accurate wave simulation has led to major advances in theory and numerical methods. In recent years, an increasing number of *conforming* space-time methods have been proposed and are actively being developed. In this context, two key properties are typically sought: unconditional stability and optimal convergence rates. Here is a brief overview of these methods.

- 1) **Second-order-in-time methods**, in which the wave field is the sole unknown:
  - 1.a) with double integration by parts in space and time (ultra-weak formulation)
    - $\circ$ [21]: Petrov-Galerkin methods with trial spaces selected to ensure optimal inf–sup stability, following a DPG approach [8]; $H^2$-regularity in both space and time is required for the test functions;
  - 1.b) with single integration by parts in space and time (weak formulation)
    - $\circ$ [28, 32]: Galerkin methods employing continuous piecewise polynomials in time, stabilized by inserting a projection in time into discontinuous polynomials of one degree lower within the grad-grad term;
    - $\circ$ [13, 16]: Galerkin methods employing maximal regularity splines in time, stabilized through the addition of a non-consistent penalty term;
    - $\circ$ [7]: coercive Galerkin methods, in which Morawetz multipliers are employed as test functions; $H^2$-regularity in both space and time is required for the trial and test functions;
    - $\circ$ [25]: a Galerkin method with continuous piecewise linear functions in time, modified by a transformation operator known as modified Hilbert transform (see [29]) to guarantee stability;
    - $\circ$ [23]: a least-squares method with continuous piecewise linear functions on unstructured meshes;
  - 1.c) with integration by parts in space but not in time (weak formulation in space)
    - $\circ$ [17]: time-stepping-like schemes (discontinuous test functions in time) employing continuous piecewise polynomials for the trial functions and exponential weights in time;
    - $\circ$ [9, 31]: time-stepping-like schemes employing continuous piecewise polynomials for the trial functions;
    - $\circ$ [15]: Galerkin methods with splines of arbitrary degree and exponential weights in time, exhibiting optimal convergence rates when the difference between polynomial degree and spline regularity is odd.
- 2) **First-order-in-time methods**, in which the unknowns are the wave field and its velocity:
  - $\circ$ [6, 18, 20]: time-stepping-like schemes employing continuous piecewise polynomials in time for the trial functions;
  - $\circ$ [14]: Galerkin methods employing maximal regularity splines in time for the trial functions, and their derivative space for the test functions;
  - $\circ$ [19]: first-order least-squares methods in both time and space;
  - $\circ$ [2, 3]: Petrov-Galerkin collocation methods in time employing arbitrary regularity splines as trial functions.

In several of the above references, discrete space–time tensor product spaces are employed. As this brief overview shows, regularity assumptions in space or time are often required, and unconditional stability (or convergence) is often proven for a particular type of time-discrete spaces (e.g. polynomials or maximal regularity splines).

In this work, we consider conforming first-order-in-time space–time methods that extend the schemes in [18, 20] to general tensor-product discrete spaces. In particular, our approach also includes spline functions in time, thus going beyond piecewise-continuous polynomial approximations.

The core idea is to exploit the exponential weights in time similarly to [31], and establish an inf–sup condition for the continuous variational formulation in suitable norms, under the assumption that the test functions are time derivatives of the trial functions. A slightly stronger solution regularity than the minimal one is required, as, e.g., in [7, 15, 19]. While in [31] the exponential weights are used only in the analysis, we keep them in the definition of the numerical method, as in [17] and then in [15]. The stability analysis of the *discrete* formulation is a straightforward extension of that of the continuous formulation, based on the same assumption that the test functions are time derivatives of the trial functions, allowing flexibility in the choice of the (conforming) trial space.

The analysis we propose shares several ideas with that in [15], which also employs exponential weights but within a second-order-in-time formulation. The main difference lies in the derivation of optimal error rates for more general choices of the discrete spaces in time. In fact, while the method in [15] is coercive, optimality in time there is guaranteed only under specific conditions.

As a final note, we emphasize that our inf-sup analysis makes use of Newton potentials and discrete Newton potential operators, thereby inducing different continuous and discrete norms, in analogy with the analysis of space–time methods for parabolic problems in [27]. In this framework, such a gap between norms may spoil quasi-optimality (see [30, Remark 3.6]). In our analysis, in addition to this gap, inf–sup stability and continuity are obtained with respect to different norms, thereby precluding the application of the standard inf-sup analysis framework for the error analysis. Nevertheless, we are able to establish optimal convergence rates in standard Bochner-Sobolev norms for sufficiently smooth solutions by employing suitable elliptic projection operators.

The paper is organized as follows: in Section 2, we introduce the model problem. In Section 3, we define and analyze the exponentially-weighted variational formulation of the space–time problem, proving its inf–sup stability and well-posedness. In Section 4, we study space–time tensor product conforming discretizations, proving unconditional stability and optimal convergence rates in both the energy and $L^2$ norms, under standard approximation assumptions on the discrete spaces. Numerical experiments are presented in Section 4 to validate the theoretical findings.

## Model Problem

Let $\varOmega$ be a Lipschitz, bounded polytopal domain in $\mathbb {R}^d$ ($d = 1,2,3$), *T* a positive final time, and $Q_T:= \varOmega \times (0,T)$ the space–time cylinder.

Given initial data $U_0 \in H_0^1(\varOmega )$, $V_0 \in L^2(\varOmega )$ and a forcing term $F \in L^2(Q_T)$, consider the following problem: find $U:\overline{Q}_T \rightarrow \mathbb {R}$ such that

1 $$\begin{aligned} {\left\{ \begin{array}{ll} \partial _t^2 U(\boldsymbol{x},t) - \nabla _{\boldsymbol{x}}\cdot (c^2(\boldsymbol{x})\nabla _{\boldsymbol{x}} U(\boldsymbol{x},t)) = F(\boldsymbol{x},t) &  (\boldsymbol{x},t) \in Q_T, \\ U(\boldsymbol{x},t) = 0 &  (\boldsymbol{x},t) \in \partial \varOmega \times (0,T], \\ U(\boldsymbol{x},0) = U_0(\boldsymbol{x}), \quad \partial _t U(\boldsymbol{x},0) = V_0(\boldsymbol{x}) &  \boldsymbol{x}\in \varOmega . \end{array}\right. } \end{aligned}$$

Here, $c = c(\boldsymbol{x})$ is the wave velocity, for which we assume, for a constant $c_0$,

2 $$\begin{aligned} c \in L^\infty (\varOmega ) \quad \text {and} \quad c(\boldsymbol{x}) \ge c_0 > 0 \quad \text {for almost every~} \boldsymbol{x}\in \varOmega . \end{aligned}$$

### Remark 1

In this paper, we restrict to homogeneous Dirichlet boundary conditions in space. We remark that Neumann boundary conditions can also be considered without substantially affecting the following analysis. In contrast, impedance boundary conditions, which couple the time derivative of *U* with its normal derivative on the boundary, require a different and more careful treatment. We leave this for future work. Nevertheless, in Section 4.3.3, we comment on how the proposed method can be extended to impedance boundary conditions and present a numerical experiment. $\blacksquare$

A well-posed variational formulation of problem (1) reads: find

$$\begin{aligned} U \in L^2(0,T; H_0^1(\varOmega )) \cap H^1(0,T; L^2(\varOmega )) \end{aligned}$$

such that $U_{|_{t=0}} = U_0$ in $H^1_0(\varOmega )$, $\partial _t U_{|_{t=0}} = V_0$ in $L^2(\varOmega )$ and, for almost every $t \in (0,T)$,

3 $$\begin{aligned} \langle \partial _t^2 U(\cdot ,t), W \rangle _{H^1_0(\varOmega )} + (c^2\nabla _{\boldsymbol{x}} U(\cdot ,t), \nabla _{\boldsymbol{x}} W)_{L^2(\varOmega )} = (F(\cdot ,t), W )_{L^2(\varOmega )} \end{aligned}$$

for all $W \in H_0^1(\varOmega )$, where $\langle \cdot ,\cdot \rangle _{H^1_0(\varOmega )}$ is the duality product between $[H_0^1(\varOmega )]'$ and $H^1_0(\varOmega )$. The solution *U* actually satisfies

4 $$\begin{aligned} U \in L^2(0,T; H_0^1(\varOmega )) \cap H^1(0,T; L^2(\varOmega )) \cap H^2(0,T;[H_0^1(\varOmega )]'); \end{aligned}$$

see, e.g., [24, Chapter 3. §8 Pag. 265].

Introducing the new unknown $V:= \partial _t U$, we rewrite problem (1) as the first-order-in-time system

5 $$\begin{aligned} {\left\{ \begin{array}{ll} \partial _t V(\boldsymbol{x},t) - \nabla _{\boldsymbol{x}}\cdot (c^2(\boldsymbol{x})\nabla _{\boldsymbol{x}} U(\boldsymbol{x},t)) = F(\boldsymbol{x},t) &  (\boldsymbol{x},t) \in Q_T, \\ \partial _t U(\boldsymbol{x},t) - V(\boldsymbol{x},t) = 0 &  (\boldsymbol{x},t) \in Q_T, \\ U(\boldsymbol{x},t) = 0 &  (\boldsymbol{x},t) \in \partial \varOmega \times (0,T), \\ U(\boldsymbol{x},0) = U_0(\boldsymbol{x}), \quad V(\boldsymbol{x},0) = V_0(\boldsymbol{x}) &  \boldsymbol{x}\in \varOmega . \end{array}\right. } \end{aligned}$$

If *U* satisfies the regularity in (4), then

$$\begin{aligned} V \in L^2(Q_T) \cap H^1(0,T; [H_0^1(\varOmega )]'). \end{aligned}$$

For the purposes of the continuous variational formulation we consider (see Section 3 below), we restrict our setting to a more regular framework and make the following assumption.

### Assumption 1

Let the wave velocity *c* be as in (2), $U_0 \in H_0^1(\varOmega )$, and

$$\begin{aligned} \begin{aligned}&F \in H^1(0,T;L^2(\varOmega )), \quad V_0 \in H^1_0(\varOmega ), \\  &F(\cdot ,0) \in L^2(\varOmega ), \quad \nabla _{\boldsymbol{x}} \cdot (c^2 \nabla _{\boldsymbol{x}} U_0) \in L^2(\varOmega ). \end{aligned} \end{aligned}$$

Under Assumption 1, one can establish improved regularity for the unique solution to (3); see, e.g., [1, Prop. 4.4], [19, Lemma 2.7], and [11, Chapter 7.2].

### Proposition 1

Under Assumption 1, there exists a unique solution to (3) and it satisfies

$$\begin{aligned} U \in L^2(0,T; H_0^1(\varOmega )), \quad \partial _t U \in L^\infty (0,T; H_0^1(\varOmega )), \quad \partial _t^2 U \in L^\infty (0,T; L^2(\varOmega )). \end{aligned}$$

Setting $V = \partial _t U$, this in particular implies

$$\begin{aligned} U \in H^1(0,T; H_0^1(\varOmega )), \quad V \in L^2(0,T; H_0^1(\varOmega )) \cap H^1(0,T; L^2(\varOmega )). \end{aligned}$$

Proposition 1 is crucial for the analysis of our variational formulation, as the properties of the associated bilinear form alone are not enough to guarantee the existence of a continuous solution.

## Space–Time Variational Formulation

In this section, we introduce and analyze a variational formulation of (5).

We set $H_{0,\bullet }^1(0,T):= \{u \in H^1(0,T): u(0)=0\},$ and start by considering the following variational formulation: find $U \in H^1_{0,\bullet }(0,T;H^1_0(\varOmega ))$ and $V \in H^1_{0,\bullet }(0,T;L^2(\varOmega ))$ such that

6 $$\begin{aligned} {\left\{ \begin{array}{ll} (\partial _t V, \lambda )_{L^2(Q_T)} + (c^2 \nabla _{\boldsymbol{x}} U, \nabla _{\boldsymbol{x}} \lambda )_{L^2(Q_T)} &  \\   &  \hspace{-3.5cm} = (F, \lambda )_{L^2(Q_T)} - (c^2\nabla _{\boldsymbol{x}} U_0, \nabla _{\boldsymbol{x}} \lambda )_{L^2(Q_T)}, \\ (\partial _t U, \chi )_{L^2(Q_T)} - (V, \chi )_{L^2(Q_T)} = (V_0,\chi )_{L^2(Q_T)}, &  \end{array}\right. } \end{aligned}$$

for all $\lambda \in L^2(0,T;H_0^1(\varOmega ))$ and $\chi \in L^2(Q_T)$. If $(\widetilde{U},\widetilde{V})$ is a solution to (5), then $(\widetilde{U} - U_0,\, \widetilde{V} - V_0)$ satisfies (6).

In the following, we introduce a modified variational formulation that incorporates weighted scalar products in time, and we study the properties of the associated bilinear form.

### Weighted Space–Time Bilinear form and Its Properties

For functions depending solely on time, we define the weighted scalar product in $L^2(0,T)$ and its associated norm

7 $$\begin{aligned} (u,v)_{L^2_e(0,T)} := \int _0^T u(s) v(s) e^{-s/T} \textrm{d}s, \quad \quad \quad \Vert u \Vert _{L^2_e(0,T)}^2 := (u,u)_{L^2_e(0,T)}. \end{aligned}$$

The important property of this scalar product is that, for all $u, v \in H^1(0,T)$,

$$\begin{aligned} (u, \partial _t v)_{L^2_e(0,T)} = -(\partial _t u, v)_{L^2_e(0,T)} + \frac{1}{T} (u,v)_{L^2_e(0,T)} + \frac{1}{e} u(T) v(T)- u(0) v(0), \end{aligned}$$

from which it follows that

8 $$\begin{aligned} (w, \partial _t w)_{L^2_e(0,T)} = \frac{1}{2T} \Vert w \Vert _{L^2_e(0,T)}^2 + \frac{1}{2e} |w(T)|^2 \qquad \text {for all~} w \in H_{0,\bullet }^1(0,T). \end{aligned}$$

Then, for space–time functions, we denote by $(\cdot ,\cdot )_{L^2_e(Q_T)}: L^2(Q_T) \times L^2(Q_T) \rightarrow \mathbb {R}$ the weighted scalar product

$$\begin{aligned} ( \chi , \lambda )_{L^2_e(Q_T)} := \int _0^T ( \chi (\cdot ,s), \lambda (\cdot ,s) )_{L^2(\varOmega )} \, e^{-s/T} \, \textrm{d}s, \end{aligned}$$

and by $\langle \cdot , \cdot \rangle _e: L^2(0,T;[H^1_0(\varOmega )]') \times L^2(0,T;H^1_0(\varOmega )) \rightarrow \mathbb {R}$ the duality pairing

$$\begin{aligned} \langle \chi , \lambda \rangle _e := \int _0^T \langle \chi (\cdot ,s), \lambda (\cdot ,s) \rangle _{H^1_0(\varOmega )} \, e^{-s/T} \, \textrm{d}s. \end{aligned}$$

#### Remark 2

As $1/e\le e^{-t/T}\le 1$ for all $t\in [0,T]$, the weighted $L^2(0,T)$ norm is equivalent to the standard one with equivalence constants that do not depend on *T*, precisely

9 $$\begin{aligned} (1/{\sqrt{e}}) \Vert w \Vert _{L^2(0,T)} \le \Vert w \Vert _{L_e^2(0,T)} \le \Vert {w} \Vert _{L^2(0,T)} \quad \text {for all~} w \in L^2(0,T), \end{aligned}$$

which also implies

10 $$\begin{aligned} (1/{\sqrt{e}}) \Vert \chi \Vert _{L^2(Q_T)} \le \Vert \chi \Vert _{L_e^2(Q_T)} \le \Vert \chi \Vert _{L^2(Q_T)} \quad \text {for all~} \chi \in L^2(Q_T). \end{aligned}$$

$$\blacksquare $$

We define the space–time bilinear form $\mathcal {A}$ as

11 $$\begin{aligned} \begin{aligned} \mathcal {A}((U,V),(\lambda ,\chi )) :=&\langle \partial _t V, \lambda \rangle _e + (c^2\nabla _{\boldsymbol{x}} U, \nabla _{\boldsymbol{x}} \lambda )_{L^2_e(Q_T)} \\  &\quad - (\partial _t U, \chi )_{L^2_e(Q_T)} + (V, \chi )_{L^2_e(Q_T)}. \end{aligned} \end{aligned}$$

The *minimal* regularity required for $\mathcal {A}$ to be well-defined is

12 $$\begin{aligned} \begin{aligned} (U,V)&\in \bigl (L^2(0,T; H_0^1(\varOmega )) \cap H^1(0,T; L^2(\varOmega ))\bigr ) \\  &\hspace{4cm} \times \bigl (L^2(Q_T) \cap H^1(0,T; [H_0^1(\varOmega )]')\bigr ), \\ (\lambda ,\chi )&\in L^2(0,T;H_0^1(\varOmega )) \times L^2(Q_T). \end{aligned} \end{aligned}$$

As in the analysis of the space–time parabolic problem in [27], we introduce the *Newton potential* operator $\mathcal {N}^\varOmega : [H_0^1(\varOmega )]' \rightarrow H_0^1(\varOmega )$ as the inverse of $-\nabla _{\boldsymbol{x}}\cdot (c^2\nabla _{\boldsymbol{x}}): H^1_0(\varOmega ) \rightarrow [H_0^1(\varOmega )]'$:

13 $$\begin{aligned} (c^2 \nabla _{\boldsymbol{x}} \mathcal {N}^\varOmega U, \nabla _{\boldsymbol{x}} V)_{L^2(\varOmega )} = \langle U,V \rangle _{H^1_0(\varOmega )} \quad \text {for all~} V \in H_0^1(\varOmega ). \end{aligned}$$

From Lax–Milgram’s lemma, $\mathcal {N}^\varOmega$ is well-defined. Moreover, it induces a norm in $[H^1_0(\varOmega )]'$, which is equivalent to the standard one:

14 $$\begin{aligned} \begin{aligned} \Vert U \Vert _{\mathcal {N}^\varOmega }^2 := \langle U, \mathcal {N}^\varOmega U \rangle _{H_0^1(\varOmega )}&= (c^2 \nabla _{\boldsymbol{x}} \mathcal {N}^\varOmega U, \nabla _{\boldsymbol{x}} \mathcal {N}^\varOmega U)_{L^2(\varOmega )} \\  &= \Vert c \nabla _{\boldsymbol{x}} \mathcal {N}^\varOmega U \Vert ^2_{L^2(\varOmega )}. \end{aligned} \end{aligned}$$

We have the following lemma.

#### Lemma 1

For all $$U \in L^2(\varOmega )$$,

$$\begin{aligned} \Vert c \nabla _{\boldsymbol{x}} \mathcal {N}^\varOmega U\Vert _{L^2(\varOmega )} \le \frac{C_\varOmega }{c_0} \Vert U\Vert _{L^2(\varOmega )}, \end{aligned}$$

where $C_\varOmega >0$ is the constant in the Poincaré inequality

$$\begin{aligned} \Vert V\Vert _{L^2(\varOmega )}\le C_\varOmega \Vert \nabla _{\boldsymbol{x}} V\Vert _{L^2(\varOmega )} \qquad \text {for all }\, V\in H^1_0(\varOmega ), \end{aligned}$$

and $c_0$ the lower bound of *c* in (2).

#### Proof

The result is standard. We report here a proof for completeness. Let us endow $H^1_0(\varOmega )$ with the norm $\Vert \nabla _{\boldsymbol{x}}\cdot \Vert _{L^2(\varOmega )}$. By the definition of $\mathcal {N}^\varOmega$ and $\Vert \cdot \Vert _{\mathcal {N}^\varOmega }$, we have

$$\begin{aligned} \Vert c \nabla _{\boldsymbol{x}} \mathcal {N}^\varOmega U\Vert _{L^2(\varOmega )}^2 =\langle U,\mathcal {N}^\varOmega U\rangle _{H^1_0(\varOmega )} \le \Vert U\Vert _{[H^1_0(\varOmega )]'}\Vert \nabla _{\boldsymbol{x}} \mathcal {N}^\varOmega U\Vert _{L^2(\varOmega )}, \end{aligned}$$

and therefore

$$\begin{aligned} \Vert c \nabla _{\boldsymbol{x}} \mathcal {N}^\varOmega U\Vert _{L^2(\varOmega )} \le \frac{1}{c_0} \Vert U\Vert _{[H^1_0(\varOmega )]'}. \end{aligned}$$

The result follows from

$$\begin{aligned} \Vert U\Vert _{[H^1_0(\varOmega )]'}:=\sup _{0\ne V\in H^1_0(\varOmega )}\frac{\langle U,V\rangle _{H^1_0(\varOmega )}}{\Vert \nabla _{\boldsymbol{x}} V\Vert _{L^2(\varOmega )}}&= \sup _{0\ne V\in H^1_0(\varOmega )}\frac{(U,V)_{L^2(\varOmega )}}{\Vert \nabla _{\boldsymbol{x}} V\Vert _{L^2(\varOmega )}} \\  &\le C_\varOmega \Vert U\Vert _{L^2(\varOmega )}, \end{aligned}$$

where, in the last step, we applied the Cauchy-Schwarz and Poincaré inequalities. $\square$

Consider functions depending on space and time. For $U \in L^2(0,T;[H_0^1(\varOmega )]')$, we denote by $\mathcal {N}^\varOmega _eU \in L^2(0,T;H_0^1(\varOmega ))$ the function defined via

$$\begin{aligned} (c^2 \nabla _{\boldsymbol{x}} \mathcal {N}^\varOmega _eU, \nabla _{\boldsymbol{x}} V)_{L^2_e(Q_T)} = \int _0^T \langle U(\cdot ,s), V(\cdot ,s) \rangle _{H_0^1(\varOmega )} \, e^{-s/T} \textrm{d}s \end{aligned}$$

for all $V \in L^2(0,T;H_0^1(\varOmega ))$. Moreover, we define for $U \in L^2(0,T;[H_0^1(\varOmega )]')$ the norm

15 $$\begin{aligned} \begin{aligned} \Vert U \Vert _{\mathcal {N}^\varOmega _e}^2&:= \int _0^T \Vert U(\cdot ,s)\Vert ^2_{\mathcal {N}^\varOmega } \, e^{-s/T} \, \textrm{d}s \\  &= \int _0^T \Vert c\nabla _{\boldsymbol{x}} \mathcal {N}^\varOmega U(\cdot ,s)\Vert ^2_{L^2(\varOmega )} \, e^{-s/T} \, \textrm{d}s \\  &=\Vert c\nabla _{\boldsymbol{x}} \mathcal {N}^\varOmega _eU\Vert _{L^2_e(Q_T)}^2. \end{aligned} \end{aligned}$$

Using Lemma 1, we prove the following auxiliary result.

#### Lemma 2

For all $U \in L^2(Q_T)$ and $V \in L^2(0,T; [H_0^1(\varOmega )]')$, we have

$$\begin{aligned} \Vert U \Vert _{\mathcal {N}^\varOmega _e} \le \frac{C_\varOmega }{c_0} \Vert U \Vert _{L^2_e(Q_T)}\,, \quad \Vert \mathcal {N}^\varOmega _eV \Vert _{L^2_e(Q_T)} \le \frac{C_\varOmega }{c_0} \Vert V \Vert _{\mathcal {N}^\varOmega _e}, \end{aligned}$$

where $C_\varOmega >0$ is the constant in the Poincaré inequality, and $c_0$ the constant in (2).

#### Proof

For all $t \in (0,T)$, if $U(\cdot ,t) \in L^2(\varOmega )$, then $\mathcal {N}^\varOmega U(\cdot ,t) \in H^1_0(\varOmega )$. Then, using Lemma 1 we derive the first bound:

$$\begin{aligned} \begin{aligned} \Vert U\Vert ^2_{\mathcal {N}^\varOmega _e}&= \int _0^T \langle U(\cdot ,t), \mathcal {N}^\varOmega U(\cdot ,t) \rangle _{H_0^1(\varOmega )} \, e^{-t/T} \, \textrm{d}t \\  &= \int _0^T \Vert c^2 \nabla _{\boldsymbol{x}} \mathcal {N}^\varOmega U(\cdot ,t) \Vert ^2_{L^2(\varOmega )} \, e^{-t/T} \, \textrm{d}t \\  &\le \frac{C_\varOmega ^2}{c_0^2} \int _0^T \Vert U(\cdot ,t) \Vert ^2_{L^2(\varOmega )} \, e^{-t/T} \, \textrm{d}t \\  &= \frac{C_\varOmega ^2}{c_0^2} \Vert U \Vert ^2_{L^2_e(Q_T)}. \end{aligned} \end{aligned}$$

For the second bound, if $V(\cdot ,t) \in [H^1_0(\varOmega )]'$, the Poincaré inequality implies

$$\begin{aligned} \Vert \mathcal {N}^\varOmega V(\cdot ,t) \Vert _{L^2(\varOmega )} \le C_\varOmega \Vert \nabla _{\boldsymbol{x}} \mathcal {N}^\varOmega V(\cdot ,t) \Vert _{L^2(\varOmega )}, \end{aligned}$$

from which we conclude

$$\begin{aligned} \Vert \mathcal {N}^\varOmega _eV \Vert _{L^2_e(Q_T)}^2&=\int _0^T \Vert \mathcal {N}^\varOmega V(\cdot ,t) \Vert _{L^2(\varOmega )}^2 \, e^{-t/T} \, \textrm{d}t \\  &\le \frac{C_\varOmega ^2}{c_0^2} \int _0^T \Vert c \nabla _{\boldsymbol{x}} \mathcal {N}^\varOmega V(\cdot ,t) \Vert _{L^2(\varOmega )}^2 \, e^{-t/T} \, \textrm{d}t \\  &= \frac{C_\varOmega ^2}{c_0^2} \int _0^T \langle V(\cdot ,t), \mathcal {N}^\varOmega V(\cdot ,t)\rangle _{H^1_0(\varOmega )} \, e^{-t/T} \, \textrm{d}t \\  &= \frac{C_\varOmega ^2}{c_0^2} \Vert V \Vert _{\mathcal {N}^\varOmega _e}^2. \end{aligned}$$

This completes the proof. $\square$

For (*U*, *V*) and $(\lambda ,\chi )$ with regularity as in (12), we define the following norms:

16 $$\begin{aligned} \begin{aligned} \Vert (U,V) \Vert ^2_{\mathcal {V}_e(Q_T)}&:= \Vert \partial _t U \Vert _{L^2_e(Q_T)}^2 + \Vert \partial _t V \Vert ^2_{\mathcal {N}^\varOmega _e} \\  &\quad + \Vert c \nabla _{\boldsymbol{x}} U \Vert ^2_{L^2_e(Q_T)} + \Vert V \Vert ^2_{L^2_e(Q_T)}, \\ \Vert (\lambda ,\chi ) \Vert ^2_{\mathcal {W}_e(Q_T)}&:= \Vert \lambda \Vert ^2_{L^2_e(Q_T)} + \Vert \chi \Vert ^2_{\mathcal {N}^\varOmega _e}. \end{aligned} \end{aligned}$$

For functions with additional regularity, the bilinear form $\mathcal {A}$ defined in (11) satisfies an inf–sup condition with respect to the norms in (16), with an inf–sup constant depending on *T*.

#### Proposition 2

(inf–sup in the norms in (16)) For any

17 $$\begin{aligned} (U,V) \in H^1_{0,\bullet }(0,T;H_0^1(\varOmega )) \times H^1_{0,\bullet }(0,T;L^2(\varOmega )), \end{aligned}$$

there exists

18 $$\begin{aligned} (\lambda ,\chi ) \in L^2(0,T;H_0^1(\varOmega )) \times L^2(Q_T) \end{aligned}$$

such that

19 $$\begin{aligned} \frac{\mathcal {A}((U,V),(\lambda ,\chi ))}{\Vert (U,V) \Vert _{\mathcal {V}_e(Q_T)}\Vert (\lambda ,\chi ) \Vert _{\mathcal {W}_e(Q_T)}} \ge \frac{1}{2\sqrt{C_\varOmega ^2/c_0^2+4T^2}}, \end{aligned}$$

where $C_\varOmega$ is the Poincaré inequality, and $c_0$ the constant in (2). Equivalently,

$$\begin{aligned} \inf _{(U,V)} \sup _{0\ne (\lambda ,\chi )}\frac{\mathcal {A}((U,V),(\lambda ,\chi ))}{\Vert (U,V) \Vert _{\mathcal {V}_e(Q_T)}\Vert (\lambda ,\chi ) \Vert _{\mathcal {W}_e(Q_T)}}\ge \frac{1}{2\sqrt{C_\varOmega ^2/c_0^2+4T^2}}, \end{aligned}$$

where the $\inf$ and $\sup$ are in the spaces in (17) and (18), respectively.

#### Proof

We prove that, for any (*U*, *V*) with regularity as in (17), there exists $(\chi , \lambda )$ with regularity as in (18) such that

20 $$\begin{aligned} \Vert ( \lambda , \chi )\Vert _{\mathcal {W}_e(Q_T)}&\le C_1 \Vert (U,V) \Vert _{\mathcal {V}_e(Q_T)}, \end{aligned}$$

21 $$\begin{aligned} \mathcal {A}((U,V),(\lambda ,\chi ))&\ge C_2\Vert (U,V) \Vert ^2_{\mathcal {V}_e(Q_T)}, \end{aligned}$$

with constants $C_1,C_2>0$ possibly depending on *T*. Then, the estimate in (19) follows with constant on the right-hand side smaller than or equal to $C_2/C_1$.

Let us fix (*U*, *V*) with regularity as in (17) and define $(\chi , \lambda )$ as

22 $$\begin{aligned} \lambda := \mathcal {N}^\varOmega _e\partial _t V + 2T\, \partial _t U, \quad \quad \chi := -\partial _t U +2T\, \partial _t V. \end{aligned}$$

Note that $(\lambda ,\chi )$ satisfies the regularity in (18). Using Lemma 2, we have

$$\begin{aligned}&\Vert (\lambda ,\chi ) \Vert ^2_{\mathcal {W}_e(Q_T)} \\  &\quad \quad = \Vert \lambda \Vert _{L^2_e(Q_T)}^2 + \Vert \chi \Vert ^2_{\mathcal {N}^\varOmega _e} \\  &\quad \quad \le \Vert \mathcal {N}^\varOmega _e\partial _t V \Vert _{L^2_e(Q_T)}^2 + 4T^2 \Vert \partial _t U \Vert _{L^2_e(Q_T)}^2 + \Vert \partial _t U\Vert ^2_{\mathcal {N}^\varOmega _e} + 4T^2 \Vert \partial _t V\Vert ^2_{\mathcal {N}^\varOmega _e} \\  &\quad \quad \le (C_\varOmega ^2/c_0^2+4T^2) \Vert (U,V) \Vert ^2_{\mathcal {V}_e(Q_T)}\,, \end{aligned}$$

which proves (20) with $C_1^2=C_\varOmega ^2/c_0^2+4T^2$. Using (8), we compute

23 $$\begin{aligned} \begin{aligned}&\mathcal {A}((U,V), (\lambda , \chi )) \\  &\quad = \Vert \partial _t V \Vert _{\mathcal {N}^\varOmega _e}^2 + 2T (\partial _t V, \partial _t U)_{L^2_e(Q_T)} + (U, \partial _t V)_{L^2_e(Q_T)} \\  &\quad \quad + 2T (c^2 \nabla _{\boldsymbol{x}} U,\nabla _{\boldsymbol{x}} \partial _t U)_{L^2_e(Q_T)} + \Vert \partial _t U \Vert _{L^2_e(Q_T)}^2 \\  &\quad \quad - 2T (\partial _t U, \partial _t V)_{L^2_e(Q_T)} - (V, \partial _t U)_{L^2_e(Q_T)} + 2T (V, \partial _t V)_{L^2_e(Q_T)} \\  &\quad = \Vert \partial _t U \Vert _{L^2_e(Q_T)}^2 + \Vert \partial _t V \Vert _{\mathcal {N}^\varOmega _e}^2 +\Vert c\nabla _{\boldsymbol{x}} U \Vert _{L^2_e(Q_T)}^2 \\  &\quad \quad + \frac{T}{e} \Vert c\nabla _{\boldsymbol{x}} U(\cdot ,T)\Vert _{L^2(\varOmega )}^2 +\Vert V \Vert _{L^2_e(Q_T)}^2 \\  &\quad \quad + \frac{T}{e}\Vert V(\cdot ,T)\Vert _{L^2(\varOmega )}^2 + (U, \partial _t V)_{L^2_e(Q_T)} - (V, \partial _t U)_{L^2_e(Q_T)}. \end{aligned} \end{aligned}$$

Recalling the definition of $\mathcal {N}^\varOmega$ in (13) and using the Young inequality, we deduce

$$\begin{aligned} (U,\partial _t V)_{L^2_e(Q_T)}&= \int _0^T ( U(\cdot ,t), \partial _t V(\cdot ,t) )_{L^2(\varOmega )} \, e^{-t/T} \, \textrm{d}t \\  &= \int _0^T (c^2\nabla _{\boldsymbol{x}} U(\cdot ,t), \nabla _{\boldsymbol{x}} \mathcal {N}^\varOmega \partial _t V(\cdot ,t) )_{L^2(\varOmega )} \, e^{-t/T} \, \textrm{d}t \\  &\ge - \frac{1}{2} \Vert c\nabla _{\boldsymbol{x}} U \Vert ^2_{L^2_e(Q_T)} - \frac{1}{2} \Vert c \nabla _{\boldsymbol{x}} \mathcal {N}^\varOmega _e\partial _t V \Vert ^2_{L^2_e(Q_T)}\\&= - \frac{1}{2} \Vert c\nabla _{\boldsymbol{x}} U \Vert ^2_{L^2_e(Q_T)} - \frac{1}{2} \Vert \partial _t V \Vert ^2_{\mathcal {N}^\varOmega _e}. \end{aligned}$$

From this and $-(V,\partial _t U)_{L^2_e(Q_T)}\ge -\frac{1}{2} \Vert V \Vert ^2_{L^2_e(Q_T)} -\frac{1}{2} \Vert \partial _t U \Vert ^2_{L^2_e(Q_T)}$, we continue (23) as

$$\begin{aligned} \mathcal {A}((U,V), (\lambda , \chi ))&\ge \Vert \partial _t U \Vert _{L^2_e(Q_T)}^2 + \Vert \partial _t V \Vert _{\mathcal {N}^\varOmega _e}^2 +\Vert c\nabla _{\boldsymbol{x}} U \Vert _{L^2_e(Q_T)}^2\ \\  &\quad +\Vert V \Vert _{L^2_e(Q_T)}^2 -\frac{1}{2} \Vert c \nabla _{\boldsymbol{x}} U \Vert _{L^2_e(Q_T)}^2 \\  &\quad - \frac{1}{2} \Vert \partial _t V \Vert _{\mathcal {N}^\varOmega _e}^2 -\frac{1}{2}\Vert V \Vert _{L^2_e(Q_T)}^2 - \frac{1}{2}\Vert \partial _t U \Vert _{L^2_e(Q_T)}^2 \\  &= \frac{1}{2} \Vert (U,V)\Vert ^2_{\mathcal {V}_e(Q_T)}. \end{aligned}$$

This leads to (21) with $C_2 = 1/2$. Thus, taking into account that we have proven (20) with $C_1=\sqrt{C_\varOmega ^2/c_0^2+4T^2}$, we obtain (19). $\square$

#### Remark 3

The additional regularity assumed in (17) gives the possibility of including the second terms in the definitions of $\chi$ and $\lambda$ in (22). Indeed, under only the minimal regularity assumption on (*U*, *V*) given in (12), the terms $\partial _t V$ and $\partial _t U$ are not guaranteed to be in $L^2(Q_T)$ and $L^2(0,T;H_0^1(\varOmega ))$, which is required by (18). $\blacksquare$

We also derive the following continuity and coercivity-like estimates.

#### Proposition 3

For (*U*, *V*) and $(\lambda ,\chi )$ with regularity as in (12), we have

$$\begin{aligned} \mathcal {A}((U,V),(\lambda ,\chi )) \le \sqrt{2} \Vert (U,V) \Vert _{\mathcal {V}_e(Q_T)}(\Vert c\nabla _{\boldsymbol{x}} \lambda \Vert _{L^2_e(Q_T)}^2+\Vert \chi \Vert _{L^2_e(Q_T)}^2)^{\frac{1}{2}}, \end{aligned}$$

where $\Vert \cdot \Vert _{\mathcal {V}_e(Q_T)}$ is the norm defined in (16). Furthermore, for (*U*, *V*) with regularity as in (12) we also have

$$\begin{aligned} \mathcal {A}((U,V),(\partial _t U, \partial _t V)) \ge \frac{1}{2T} ( \Vert c\nabla _{\boldsymbol{x}} U \Vert ^2_{L^2_e(Q_T)} + \Vert V \Vert ^2_{L^2_e(Q_T)}). \end{aligned}$$

#### Proof

The continuity property follows from the definition of $\mathcal {N}^\varOmega$ in (13) and the Cauchy-Schwarz inequality:

$$\begin{aligned} \Vert {\mathcal {A}((U,V),(\lambda ,\chi ))}\Vert&\le \Vert \partial _t V \Vert _{\mathcal {N}^\varOmega _e} \Vert c \nabla _{\boldsymbol{x}} \lambda \Vert _{L^2_e(Q_T)} +\Vert c \nabla _{\boldsymbol{x}} U \Vert _{L^2_e(Q_T)} \Vert c \nabla _{\boldsymbol{x}} \lambda \Vert _{L^2_e(Q_T)} \\  &\qquad + \Vert \partial _t U \Vert _{L^2_e(Q_T)}\Vert \chi \Vert _{L^2_e(Q_T)}+\Vert V \Vert _{L^2_e(Q_T)}\Vert \chi \Vert _{L^2_e(Q_T)} \\  &\le \sqrt{2} \Vert (U,V) \Vert _{\mathcal {V}_e(Q_T)}(\Vert c\nabla _{\boldsymbol{x}} \lambda \Vert _{L^2_e(Q_T)}^2+\Vert \chi \Vert _{L^2_e(Q_T)}^2)^{\frac{1}{2}}. \end{aligned}$$

To prove the coercivity-like property, we apply (8):

$$\begin{aligned} \mathcal {A}((U,V),(\partial _t U,\partial _t V))&= (\partial _t V, \partial _t U)_{L^2_e(Q_T)} + (c^2\nabla _{\boldsymbol{x}} U, \nabla _{\boldsymbol{x}} \partial _t U)_{L^2_e(Q_T)} \\  &\qquad -(\partial _t U, \partial _t V)_{L^2_e(Q_T)} + (V,\partial _t V)_{L^2_e(Q_T)} \\  &= (c^2 \nabla _{\boldsymbol{x}} U, \nabla _{\boldsymbol{x}} \partial _t U)_{L^2_e(Q_T)} + (V,\partial _t V)_{L^2_e(Q_T)} \\  &\ge \frac{1}{2T} (\Vert c \nabla _{\boldsymbol{x}} U \Vert _{L^2_e(Q_T)}^2 + \Vert V \Vert _{L^2_e(Q_T)}^2). \end{aligned}$$

This completes the proof. $\square$

#### Remark 4

We emphasize that, for the bilinear form $\mathcal {A}$, we established continuity using a stronger norm for the test functions, as compared to the norm employed in the inf–sup condition (19), so that the Nečas theorem does not apply. This is why we adopt Assumption 1, which guarantees the existence of a solution to the strong problem (Proposition 1), and consequently to formulation (6). $\blacksquare$

### Space–Time Variational Formulation

Finally, we consider the variational formulation 
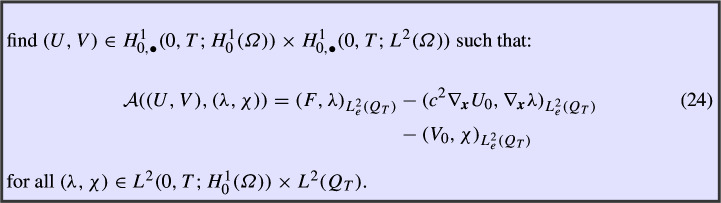
 As a consequence of Remark 4, for the analysis of problem (24), Nečas’ theorem cannot be applied, and we rely on the existence result in Proposition 1.

#### Theorem 2

Let us assume the regularity on the data as in Assumption 1. Then, there exists a unique solution to problem (24), and it satisfies

$$\begin{aligned} \begin{aligned} \Vert (U,V) \Vert _{\mathcal {V}_e(Q_T)} \le 2\sqrt{C_\varOmega ^2/c_0^2 + 4T^2}\, \bigl (\Vert F\Vert _{L^2_e(Q_T)}&+\sqrt{T}\,\Vert \nabla _{\boldsymbol{x}} \cdot (c^2 \nabla _{\boldsymbol{x}} U_0)\Vert _{L^2(\varOmega )} \\  &+\sqrt{T}\, \Vert c\nabla _{\boldsymbol{x}} V_0 \Vert _{L^2(\varOmega )}\bigr ). \end{aligned} \end{aligned}$$

Moreover, it holds true that $V \in L^2(0,T;H_0^1(\varOmega ))$.

#### Proof

The existence of a solution *U* to the variational formulation (3), with the required regularity, follows from Assumption 1, see Proposition 1. Setting $V = \partial _t U$, this implies the existence of a solution to (24), together with the property $V \in L^2(0,T;H_0^1(\varOmega ))$. The corresponding stability estimate follows from the inf–sup condition in Proposition 2, together with the following continuity property of the functional on the right-hand side:

$$\begin{aligned} \begin{aligned}&(F,\lambda )_{L^2_e(Q_T)} - (c^2 \nabla _{\boldsymbol{x}} U_0, \nabla _{\boldsymbol{x}} \lambda )_{L^2_e(Q_T)} - (V_0,\chi )_{L^2_e(Q_T)} \\  &\quad =(F,\lambda )_{L^2_e(Q_T)} + (\nabla _{\boldsymbol{x}}\cdot (c^2 \nabla _{\boldsymbol{x}}U_0), \lambda )_{L^2_e(Q_T)} - (c\nabla _{\boldsymbol{x}} V_0,c\nabla _{\boldsymbol{x}} \mathcal {N}^\varOmega _e\chi )_{L^2_e(Q_T)} \\  &\quad \le \Big (\Vert F \Vert _{L^2_e(Q_T)} +\sqrt{T}\,\Vert \nabla _{\boldsymbol{x}} \cdot (c^2 \nabla _{\boldsymbol{x}} U_0)\Vert _{L^2(\varOmega )} \\  &\qquad +\sqrt{T}\, \Vert c\nabla _{\boldsymbol{x}} V_0 \Vert _{L^2(\varOmega )}\Big ) \Vert (\lambda ,\chi ) \Vert _{\mathcal {W}_e(Q_T)}, \end{aligned} \end{aligned}$$

where, in the last step, we applied the Cauchy-Schwarz inequality and used the identity $\Vert c\nabla _{\boldsymbol{x}} \mathcal {N}^\varOmega _e\chi \Vert _{L^2_e(Q_T)}=\Vert \chi \Vert _{\mathcal {N}^\varOmega _e}$, see (15). Indeed, we compute

$$\begin{aligned}&\frac{1}{2\sqrt{C_\varOmega ^2/c_0^2+4T^2}} \Vert (U,V) \Vert _{\mathcal {V}_e(Q_T)} \\  &\quad \le \sup _{0 \ne (\lambda ,\chi ) \in (L^2(0,T;H_0^1(\varOmega )) \times L^2(Q_T))}\frac{\mathcal {A}((U,V),(\lambda ,\chi ))}{\Vert (\lambda ,\chi ) \Vert _{\mathcal {W}_e(Q_T)}} \\  &\quad \le \Vert F\Vert _{L^2_e(Q_T)} +\sqrt{T}\,\Vert \nabla _{\boldsymbol{x}} \cdot (c^2 \nabla _{\boldsymbol{x}} U_0)\Vert _{L^2(\varOmega )} +\sqrt{T}\, \Vert c\nabla _{\boldsymbol{x}} V_0 \Vert _{L^2(\varOmega )}. \end{aligned}$$

This proves the stability estimate. For the uniqueness of the solution, by linearity, it is enough to consider the case of $F=0$ and $U_0=V_0=0$, and prove that the solution is zero. From the stability estimate and the fact that $\mathcal {V}_e(Q_T)$ is a norm in $H^1_{0,\bullet }(0,T;H_0^1(\varOmega )) \times H^1_{0,\bullet }(0,T;L^2(\varOmega ))$, we have that $U=V=0$, and the proof is complete. $\square$

## Discretization of the Space–Time Problem

In this section, we study conforming space–time tensor product discretizations of the variational formulation in (24), proving their unconditional stability and deriving error estimates.

### Definition of the Method and Well-Posedness

For the spatial discretization, let us consider a discrete space $S_{h_{\boldsymbol{x}}}(\varOmega ) \subset H_0^1(\varOmega )$ depending on a spatial parameter $h_{\boldsymbol{x}}$ (e.g. piecewise linear, continuous functions over a triangulation of $\varOmega$ of mesh size $h_{\boldsymbol{x}}$). For the temporal discretization, we introduce a discrete space $S_{h_t}(0,T) \subset H_{0,\bullet }^1(0,T)$ on a mesh of (0, *T*) with mesh size $h_t$. Finally, we define the tensor product spaces

$$\begin{aligned} Q_{\boldsymbol{h}}(Q_T):=S_{h_{\boldsymbol{x}}}(\varOmega ) \otimes S_{h_t}(0,T), \quad \quad \partial _t Q_{\boldsymbol{h}} (Q_T) :=S_{h_{\boldsymbol{x}}}(\varOmega ) \otimes ~\partial _t S_{h_t}(0,T). \end{aligned}$$

The conforming discretization of (24) we consider reads as follows: 
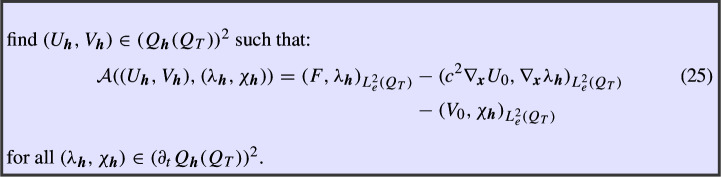


#### Remark 5

We observe that the discrete spaces for $V_{\boldsymbol{h}}$ and $\chi _{\boldsymbol{h}}$ are more regular in space than what is strictly required by (24). The choice to employ the same discretization in space for both *U* and *V* follows the approach of [18], which is the starting point of our investigation. Alternatively, one could consider replacing $S_{h_{\boldsymbol{x}}}(\varOmega )$ in the space discretization of *V* and $\chi$ with a subspace of $L^2(\varOmega )$, admitting discontinuities. While the stability analysis extends to this setting with only minor modifications, the error analysis requires a different approach to avoid applying the elliptic projection to $V_{\boldsymbol{h}}$. We have chosen to retain the current discretization for consistency with the existing literature and to develop the complete analysis within this framework. $\blacksquare$

Let $\mathcal {N}^\varOmega _{h_{\boldsymbol{x}}}: L^2(\varOmega ) \rightarrow S_{h_{\boldsymbol{x}}}(\varOmega )$ be the *discrete* Newton potential operator defined by

26 $$\begin{aligned} (c^2 \nabla _{\boldsymbol{x}} \mathcal {N}^\varOmega _{h_{\boldsymbol{x}}}U, \nabla _{\boldsymbol{x}} V_{h_{\boldsymbol{x}}})_{L^2(\varOmega )} = (U,V_{h_{\boldsymbol{x}}})_{L^2(\varOmega )} \quad \text {for all~} V_{h_{\boldsymbol{x}}} \in S_{h_{\boldsymbol{x}}}(\varOmega ). \end{aligned}$$

Owing to Lax–Milgram’s lemma, the operator $\mathcal {N}^\varOmega _{h_{\boldsymbol{x}}}$ is well-defined. Proceeding similarly to Lemma 1, we take $V_{h_{\boldsymbol{x}}}=\mathcal {N}^\varOmega _{h_{\boldsymbol{x}}}U$ in (26) and, using the Cauchy–Schwarz and Poincaré inequalities, we obtain

$$\begin{aligned} \begin{aligned} \Vert c \nabla _{\boldsymbol{x}} \mathcal {N}^\varOmega _{h_{\boldsymbol{x}}}U \Vert _{L^2(\varOmega )}^2&=(U,\mathcal {N}^\varOmega _{h_{\boldsymbol{x}}}U)_{L^2(\varOmega )} \\  &\le \Vert U\Vert _{L^2(\varOmega )} \Vert \mathcal {N}^\varOmega _{h_{\boldsymbol{x}}}U\Vert _{L^2(\varOmega )} \\  &\le \frac{C_\varOmega }{c_0} \Vert U \Vert _{L^2(\varOmega )}\Vert c \nabla _{\boldsymbol{x}} \mathcal {N}^\varOmega _{h_{\boldsymbol{x}}}U \Vert _{L^2(\varOmega )}. \end{aligned} \end{aligned}$$

From this, we deduce for all $U \in L^2(\varOmega )$

$$\begin{aligned} \Vert c \nabla _{\boldsymbol{x}} \mathcal {N}^\varOmega _{h_{\boldsymbol{x}}}U \Vert _{L^2(\varOmega )} \le \frac{C_\varOmega }{c_0} \Vert U \Vert _{L^2(\varOmega )}. \end{aligned}$$

In $L^2(\varOmega )$, similarly as in (14), we define $\Vert U \Vert _{\mathcal {N}^\varOmega _{h_{\boldsymbol{x}}}}:= \Vert c \nabla _{\boldsymbol{x}} \mathcal {N}^\varOmega _{h_{\boldsymbol{x}}}U \Vert _{L^2(\varOmega )}$. Note that this is a seminorm in $L^2(\varOmega )$, and is actually a norm in $S_{h_{\boldsymbol{x}}}(\varOmega )$. Indeed, for $U_{h_{\boldsymbol{x}}} \in S_{h_{\boldsymbol{x}}}(\varOmega )$, if $\Vert U_{h_{\boldsymbol{x}}} \Vert _{\mathcal {N}^\varOmega _{h_{\boldsymbol{x}}}} = 0$, then $\mathcal {N}^\varOmega _{h_{\boldsymbol{x}}}U_{h_{\boldsymbol{x}}} = 0$ and from (26)

$$\begin{aligned} (U_{h_{\boldsymbol{x}}}, V_{h_{\boldsymbol{x}}})_{L^2(\varOmega )} = 0 \quad \text {for all~} V_{h_{\boldsymbol{x}}} \in S_{h_{\boldsymbol{x}}}(\varOmega ), \end{aligned}$$

which implies $U_{h_{\boldsymbol{x}}} =0$. Moreover, we notice that, for all $U \in L^2(\varOmega )$,

27 $$\begin{aligned} \Vert U \Vert _{\mathcal {N}^\varOmega _{h_{\boldsymbol{x}}}} \le \Vert U \Vert _{\mathcal {N}^\varOmega }. \end{aligned}$$

Indeed, with (26), (13) and Cauchy-Schwarz inequality, we compute

$$\begin{aligned} \Vert U \Vert ^2_{\mathcal {N}^\varOmega _{h_{\boldsymbol{x}}}}&= (c^2\nabla _{\boldsymbol{x}} \mathcal {N}^\varOmega _{h_{\boldsymbol{x}}}U, \nabla _{\boldsymbol{x}} \mathcal {N}^\varOmega _{h_{\boldsymbol{x}}}U)_{L^2(\varOmega )} = (U, \mathcal {N}^\varOmega _{h_{\boldsymbol{x}}}U)_{L^2(\varOmega )} \\  &= (c^2\nabla _{\boldsymbol{x}} \mathcal {N}^\varOmega U, \nabla _{\boldsymbol{x}} \mathcal {N}^\varOmega _{h_{\boldsymbol{x}}}U)_{L^2(\varOmega )} \le \Vert c\nabla _{\boldsymbol{x}} \mathcal {N}^\varOmega U \Vert _{L^2(\varOmega )} \Vert c\nabla _{\boldsymbol{x}} \mathcal {N}^\varOmega _{h_{\boldsymbol{x}}}U \Vert _{L^2(\varOmega )} \\  &= \Vert U \Vert _{\mathcal {N}^\varOmega } \Vert U \Vert _{\mathcal {N}^\varOmega _{h_{\boldsymbol{x}}}}. \end{aligned}$$

For space–time functions $U \in L^2(Q_T)$, we define $\mathcal {N}^\varOmega _{e,\boldsymbol{h}}U \in S_{h_{\boldsymbol{x}}}(\varOmega ) \otimes L^2(0,T)$ by

$$\begin{aligned} (c^2 \nabla _{\boldsymbol{x}} \mathcal {N}^\varOmega _{e,\boldsymbol{h}}U, \nabla _{\boldsymbol{x}} V_{h_{\boldsymbol{x}}})_{L^2_e(Q_T)} = \int _0^T ( U(\cdot ,s), V_{h_{\boldsymbol{x}}}(\cdot ,s) )_{L^2(\varOmega )} \, e^{-s/T} \textrm{d}s \end{aligned}$$

for all $V_{h_{\boldsymbol{x}}} \in S_{h_{\boldsymbol{x}}}(\varOmega ) \otimes L^2(0,T)$, and the seminorm in $L^2(Q_T)$ (norm in $S_{h_{\boldsymbol{x}}}(\varOmega ) \otimes L^2(0,T)$)

$$\begin{aligned} \Vert U \Vert _{\mathcal {N}^\varOmega _{e,\boldsymbol{h}}}^2 := \int _0^T \Vert c \nabla _{\boldsymbol{x}} \mathcal {N}^\varOmega _{h_{\boldsymbol{x}}}U(\cdot ,s) \Vert _{L^2(\varOmega )}^2 \, e^{-s/T} \, \textrm{d}s. \end{aligned}$$

We summarize in the following lemma auxiliary results analogous to those in Lemma 2.

#### Lemma 3

For all $U, V\in L^2(Q_T)$, we have

$$\begin{aligned} \Vert U \Vert _{\mathcal {N}^\varOmega _{e,\boldsymbol{h}}} \le \frac{C_\varOmega }{c_0} \Vert U \Vert _{{L^2_e(Q_T)}}\, , \quad \Vert \mathcal {N}^\varOmega _{e,\boldsymbol{h}}V \Vert _{{L^2_e(Q_T)}} \le \frac{C_\varOmega }{c_0} \Vert V \Vert _{\mathcal {N}^\varOmega _{e,\boldsymbol{h}}}, \end{aligned}$$

where $C_\varOmega >0$ is the constant in the Poincaré inequality, and $c_0$ the constant in (2).

For $(U_{\boldsymbol{h}}, V_{\boldsymbol{h}}) \in (Q_{\boldsymbol{h}}(Q_T))^2$ and $(\lambda _{\boldsymbol{h}}, \chi _{\boldsymbol{h}}) \in (\partial _t Q_{\boldsymbol{h}}(Q_T))^2$, we define the following discrete norms

28 $$\begin{aligned} \begin{aligned} \Vert (U_{\boldsymbol{h}},V_{\boldsymbol{h}}) \Vert ^2_{\mathcal {V}_{e,\boldsymbol{h}}(Q_T)}&:= \Vert \partial _t U_{\boldsymbol{h}} \Vert _{{L^2_e(Q_T)}}^2 + \Vert \partial _t V_{\boldsymbol{h}} \Vert ^2_{\mathcal {N}^\varOmega _{e,\boldsymbol{h}}} \\  &\quad \quad \quad \quad \quad \quad \quad + \Vert c \nabla _{\boldsymbol{x}} U_{\boldsymbol{h}} \Vert ^2_{{L^2_e(Q_T)}} + \Vert V_{\boldsymbol{h}} \Vert ^2_{{L^2_e(Q_T)}}, \\ \Vert (\lambda _{\boldsymbol{h}},\chi _{\boldsymbol{h}}) \Vert ^2_{\mathcal {W}_{e,\boldsymbol{h}}(Q_T)}&:= \Vert \lambda _{\boldsymbol{h}} \Vert ^2_{{L^2_e(Q_T)}} + \Vert \chi _{\boldsymbol{h}} \Vert ^2_{\mathcal {N}^\varOmega _{e,\boldsymbol{h}}}. \end{aligned} \end{aligned}$$

#### Proposition 4

(inf–sup in the discrete norms (28)) For all $(U_{\boldsymbol{h}},V_{\boldsymbol{h}}) \in (Q_{\boldsymbol{h}}(Q_T))^2$ there exists $(\lambda _{\boldsymbol{h}},\chi _{\boldsymbol{h}}) \in (\partial _t Q_{\boldsymbol{h}}(Q_T))^2$ such that

$$\begin{aligned} \frac{\mathcal {A}((U_{\boldsymbol{h}},V_{\boldsymbol{h}}),(\lambda _{\boldsymbol{h}},\chi _{\boldsymbol{h}}))}{\Vert (U_{\boldsymbol{h}},V_{\boldsymbol{h}}) \Vert _{\mathcal {V}_{e,\boldsymbol{h}}(Q_T)}\Vert (\lambda _{\boldsymbol{h}},\chi _{\boldsymbol{h}}) \Vert _{\mathcal {W}_{e,\boldsymbol{h}}(Q_T)}} \ge \frac{1}{2\sqrt{C_\varOmega ^2/c_0^2+4T^2}} \end{aligned}$$

where $C_\varOmega$ is the constant in the Poincaré inequality.

#### Proof

The proof is identical to that of Proposition 2, using the bounds in Lemma 3. The test functions to be used here are

$$\begin{aligned} \lambda _{\boldsymbol{h}} = \mathcal {N}_{e,\boldsymbol{h}}^\varOmega \partial _t V_{\boldsymbol{h}} + 2T \partial _t U_{\boldsymbol{h}}, \quad \chi _{\boldsymbol{h}} = - \partial _t U_{\boldsymbol{h}} + 2T \partial _t V_{\boldsymbol{h}}. \end{aligned}$$

The result then follows by the same argument as in Proposition 2. $\square$

We observe that the inf–sup constant in both Propositions 2 and 4 depends on the final time *T*. To verify this dependence numerically, we computed the discrete inf–sup constants of the bilinear form $\mathcal {A}$ on the domain $Q_T = (0,1) \times (0,T)$. We employed spaces of maximal regularity B-splines of degree $p_t=p_{\boldsymbol{x}} = 4$ for both space and time, with uniform mesh sizes $h=h_t=h_{\boldsymbol{x}}$. The constants, obtained by solving a generalized eigenvalue problem for various values of *T* and *h*, are reported in Table 1. As expected from the theoretical bound in Proposition 4, the inf–sup constant is independent of the mesh size *h* but depends on *T* approximately as $\mathcal {O}(T^{-1})$.

**Table 1:** Discrete inf–sup constants of $\mathcal {A}$ with respect to the $\mathcal {V}_{e,\boldsymbol{h}}(Q_T)$ and $\mathcal {W}_{e,\boldsymbol{h}}(Q_T)$ norms using maximal regularity splines of degree $p_t = p_{\boldsymbol{x}} =4$, for varying final time *T* and mesh size *h*.

$h=h_t=h_{\boldsymbol{x}}$	$T=1$	$T=2$	$T=4$	$T=8$
0.125	1.1952	0.65109	0.33363	0.16788
0.063	1.1952	0.65109	0.33363	0.16788

#### Remark 6

For comparison, we also computed the inf–sup constants with respect to the unweighted norms, i.e., using the standard $L^2(0,T)$ inner product instead of the exponentially weighted one in (28). The corresponding results are reported in Table 2. We observe that the inf–sup constants obtained with the unweighted norms differ from those with the weighted ones roughly by a constant scaling factor. In particular, they exhibit the same behavior with respect to *h* and *T* as in the weighted case, namely independence of *h* and an approximately $\mathcal {O}(T^{-1})$ dependence on *T*.

**Table 2:** Discrete inf–sup constants of $\mathcal {A}$ with respect to the norms in (28) without exponential weights, using maximal regularity splines of degree $p_t = p_{\boldsymbol{x}}=4$, for varying final time *T* and mesh size *h*.

$h=h_t=h_{\boldsymbol{x}}$	$T=1$	$T=2$	$T=4$	$T=8$
0.125	0.67658	0.37447	0.19255	0.09696
0.063	0.67658	0.37447	0.19255	0.09696

As a consequence of Proposition 4, we have well-posedness of the discrete formulation in (25).

#### Corollary 1

Let the data satisfy $F \in L^2(Q_T), V_0 \in L^2(\varOmega )$ and $U_0$ such that $\nabla _{\boldsymbol{x}} \cdot ( c^2 \nabla _{\boldsymbol{x}} U_0) \in L^2(\varOmega )$. Then, there exists a unique solution to problem (25), and it satisfies

$$\begin{aligned} \begin{aligned} \Vert (U_{\boldsymbol{h}},V_{\boldsymbol{h}}) \Vert _{\mathcal {V}_{e,\boldsymbol{h}}(Q_T)} \le 2\sqrt{C_\varOmega ^2/c_0^2 + 4T^2} \bigl (\Vert F\Vert _{{L^2_e(Q_T)}}&+\sqrt{T}\Vert \nabla _{\boldsymbol{x}} \cdot (c^2 \nabla _{\boldsymbol{x}} U_0)\Vert _{L^2(\varOmega )} \\  &+ \sqrt{T}\Vert c\nabla _{\boldsymbol{x}} \varPi _{h_{\boldsymbol{x}}}^0 V_0\Vert _{L^2(\varOmega )}\bigr ), \end{aligned} \end{aligned}$$

where $\varPi _{h_{\boldsymbol{x}}}^0$ is the $L^2(\varOmega )$ projection operator into $S_{h_{\boldsymbol{x}}}(\varOmega )$.

#### Proof

The stability estimate is derived from the inf–sup condition of Proposition 4 similarly as in Theorem 2. The only difference is the use of $\varPi _{h_{\boldsymbol{x}}}^0$ in the continuity property of the functional on the right-hand side:

$$\begin{aligned} \begin{aligned}&(F,\lambda _{\boldsymbol{h}})_{L^2_e(Q_T)} - (c^2 \nabla _{\boldsymbol{x}} U_0, \nabla _{\boldsymbol{x}} \lambda _{\boldsymbol{h}})_{L^2_e(Q_T)} - (V_0,\chi _{\boldsymbol{h}})_{L^2_e(Q_T)} \\  &=(F,\lambda _{\boldsymbol{h}})_{L^2_e(Q_T)} + (\nabla _{\boldsymbol{x}}\cdot (c^2 \nabla _{\boldsymbol{x}}U_0), \lambda _{\boldsymbol{h}})_{L^2_e(Q_T)} - (\varPi _{h_{\boldsymbol{x}}}^0 V_0, \chi _{\boldsymbol{h}})_{L^2_e(Q_T)} \\  &=(F,\lambda _{\boldsymbol{h}})_{L^2_e(Q_T)} + (\nabla _{\boldsymbol{x}}\cdot (c^2 \nabla _{\boldsymbol{x}}U_0), \lambda _{\boldsymbol{h}})_{L^2_e(Q_T)} \\  &\hspace{4cm} - (c\nabla _{\boldsymbol{x}} \varPi _{h_{\boldsymbol{x}}}^0 V_0,c\nabla _{\boldsymbol{x}} \mathcal {N}^\varOmega _{e,\boldsymbol{h}}\chi _{\boldsymbol{h}})_{L^2_e(Q_T)} \\  &\le \Big (\Vert F \Vert _{L^2_e(Q_T)} +\sqrt{T}\,\Vert \nabla _{\boldsymbol{x}} \cdot (c^2 \nabla _{\boldsymbol{x}} U_0)\Vert _{L^2(\varOmega )} \\  &\hspace{4cm} +\sqrt{T}\, \Vert c\nabla _{\boldsymbol{x}} \varPi _{h_{\boldsymbol{x}}}^0 V_0 \Vert _{L^2(\varOmega )}\Big ) \Vert (\lambda _{\boldsymbol{h}},\chi _{\boldsymbol{h}}) \Vert _{\mathcal {W}_{e,\boldsymbol{h}}(Q_T)}. \end{aligned} \end{aligned}$$

The uniqueness of the solution follows from the stability estimate, and the existence follows from the uniqueness and the fact that (25) is a square, finite dimensional algebraic linear system. $\square$

#### Remark 7

We note that the stability of the discrete problem (25) requires less regularity of the data compared to that of the continuous problem (24), which depends on Assumption 1. $\blacksquare$

#### Remark 8

The use of exponential weights allows us to prove stability for a general class of conforming tensor product discrete spaces, under the minimal assumption that the trial space is the time derivative of the test space. From a practical point of view, the presence of the exponential weights in the discrete formulation may be unnecessary. This is the case for continuous piecewise polynomial approximations in time [9, 18, 20, 31], where discrete stability is proven by selecting test functions containing a weight that mimics an exponential function. Whether this strategy can be extended to more general discrete spaces remains an open question.

For general conforming tensor product discrete spaces where the trial space is the time derivative of the test space, our analysis framework does not extend to the formulation without any weighting. Therefore, it is not yet clear whether such an extension would incur any loss in convergence or stability. Moreover, identifying specific discretization spaces in time where the exponential weight is truly essential in practice would provide valuable insight. Preliminary numerical experiments suggest that this is not the case using splines, where removing the weight does not significantly affect the results, see the numerical test reported in Section 4.3.4 below. Follow-up on these open questions would be an interesting direction for future investigations. $\blacksquare$

### Error Estimates

In this section, we derive error estimates for the solution of (25) in Bochner-Sobolev norms with optimal convergence rates as in [18, 20].

In this analysis, we use similar techniques as in [9] and [20]. We start by introducing projection operators and their properties in Section 4.2.1, then we derive convergence rates in Section 4.2.2 under standard assumptions on the discrete spaces.

#### Projection Operators

We introduce the following projection operators:

- (elliptic projector in space) $\varPi ^{\nabla }_{h_{\boldsymbol{x}}}: L^2(0,T;H^1(\varOmega )) \rightarrow S_{h_{\boldsymbol{x}}}(\varOmega ) \otimes L^2(0,T)$ defined by
  29 $$\begin{aligned} (c^2 \nabla _{\boldsymbol{x}} (\varPi ^{\nabla }_{h_{\boldsymbol{x}}}- {{\,\textrm{Id}\,}}) W, \nabla _{\boldsymbol{x}} W_{h_{\boldsymbol{x}}})_{L_e^2(Q_T)} = 0 \end{aligned}$$
  for all $$W_{h_{\boldsymbol{x}}} \in S_{h_{\boldsymbol{x}}}(\varOmega ) \otimes L^2(0,T)$$,
- (elliptic projector in time) $\varPi _{h_t}^{\partial _t}: H^1(0,T;L^2(\varOmega )) \rightarrow L^2(\varOmega ) \otimes S_{h_t}(0,T)$ defined by
  30 $$\begin{aligned} (\partial _t (\varPi _{h_t}^{\partial _t}-{{\,\textrm{Id}\,}}) W, \chi _{h_t})_{L^2_e(Q_T)} = 0 \quad \end{aligned}$$
  for all $$\chi _{h_t} \in L^2(\varOmega ) \otimes \partial _t S_{h_t}(0,T)$$.

##### Remark 9

The discrete Newton potential in (26) can be equivalently defined as the elliptic projection $\varPi _{h_{\boldsymbol{x}}}^{\nabla }$ of its continuous counterpart (13), i.e. $\mathcal {N}^\varOmega _{e,\boldsymbol{h}} = \varPi ^{\nabla }_{h_{\boldsymbol{x}}} \mathcal {N}_e^\varOmega$. $\blacksquare$

In the following lemma, we collect useful properties of these operators; see, e.g., [5, §2] and [28, Lemma 10]. We observe that the results of these references immediately carry over to our setting, owing to the equivalence of the weighted and standard $L^2$ norms; see Remark 2.

##### Lemma 4

The following composition of projectors is well defined and commutes:

$$\begin{aligned} \varPi ^{\nabla }_{h_{\boldsymbol{x}}}\varPi _{h_t}^{\partial _t}&= \varPi _{h_t}^{\partial _t}\varPi ^{\nabla }_{h_{\boldsymbol{x}}}: H^1(0,T;H^1(\varOmega )) \rightarrow Q_{\boldsymbol{h}}(Q_T). \end{aligned}$$

Moreover, the differential operators and projectors commute:

$$\begin{aligned} \partial _t \varPi ^{\nabla }_{h_{\boldsymbol{x}}}W = \varPi ^{\nabla }_{h_{\boldsymbol{x}}}\partial _t W, \quad \quad \nabla _{\boldsymbol{x}} \varPi _{h_t}^{\partial _t}W = \varPi _{h_t}^{\partial _t}\nabla _{\boldsymbol{x}} W \quad \quad \quad \end{aligned}$$

for all $W \in H^1(0,T;H^1(\varOmega ))$. Furthermore, the projectors satisfy standard approximation properties:

- for all $$W \in L^2(0,T;H^1(\varOmega ))$$,
  31 $$\begin{aligned} \begin{aligned}&\Vert c \nabla _{\boldsymbol{x}} (\varPi ^{\nabla }_{h_{\boldsymbol{x}}}-{{\,\textrm{Id}\,}}) W\Vert _{L_e^2(Q_T)} \\  &\quad \quad \quad \quad \quad \quad = \inf _{W_{h_{\boldsymbol{x}}} \in S_{h_{\boldsymbol{x}}}(\varOmega ) \otimes L^2(0,T)} \Vert c\nabla _{\boldsymbol{x}} (W_{h_{\boldsymbol{x}}} - W) \Vert _{L_e^2(Q_T)}; \end{aligned} \end{aligned}$$
- for all $$W \in H^1(0,T;L^2(\varOmega ))$$,
  32 $$\begin{aligned} \Vert \partial _t (\varPi _{h_t}^{\partial _t}- {{\,\textrm{Id}\,}}) W \Vert _{L^2_e(Q_T)} = \inf _{W_{h_t} \in L^2(\varOmega ) \otimes S_{h_t}(0,T)} \Vert \partial _t(W_{h_t} - W) \Vert _{L_e^2(Q_T)}. \end{aligned}$$

Finally, we have the following stability estimates:

33 $$\begin{aligned} \Vert c \nabla _{\boldsymbol{x}} \varPi ^{\nabla }_{h_{\boldsymbol{x}}}W\Vert _{L_e^2(Q_T)}&\le \Vert c\nabla _{\boldsymbol{x}} W \Vert _{L_e^2(Q_T)} \quad \,\,\,\, \text {for all~} W \in L^2(0,T;H^1(\varOmega )), \end{aligned}$$

34 $$\begin{aligned} \Vert \partial _t \varPi _{h_t}^{\partial _t}W \Vert _{{L^2_e(Q_T)}}&\le \Vert \partial _t W \Vert _{{L^2_e(Q_T)}} \quad \ \ \,\,\,\,\, \text {for all~} W \in H^1(0,T;L^2(\varOmega )). \end{aligned}$$

As a consequence of the inf–sup condition established in Proposition 2, we derive the following auxiliary result.

##### Lemma 5

Assume the regularity on the data as in Assumption 1. Let (*U*, *V*) be the unique solution to (24), whose regularity is stated in Proposition 1, and let $(U_{\boldsymbol{h}},V_{\boldsymbol{h}})$ be the unique discrete solution to (25). Suppose also that

35 $$\begin{aligned} \nabla _{\boldsymbol{x}} \cdot (c^2 \nabla _{\boldsymbol{x}} U) \in H^1(0,T;L^2(\varOmega )). \end{aligned}$$

Then, it holds

$$\begin{aligned}&\Vert (\varPi ^{\nabla }_{h_{\boldsymbol{x}}}\varPi _{h_t}^{\partial _t}U- U_{\boldsymbol{h}}, \varPi ^{\nabla }_{h_{\boldsymbol{x}}}\varPi _{h_t}^{\partial _t}V - V_{\boldsymbol{h}})\Vert _{\mathcal {V}_{e,\boldsymbol{h}}(Q_T)} \\  &\quad \quad \le \beta \bigl (\Vert (\varPi ^{\nabla }_{h_{\boldsymbol{x}}}-{{\,\textrm{Id}\,}}) \partial _t V \Vert _{{L^2_e(Q_T)}} \\  &\quad \quad \quad + \Vert (\varPi _{h_t}^{\partial _t}- {{\,\textrm{Id}\,}}) \nabla _{\boldsymbol{x}} \cdot (c^2 \nabla _{\boldsymbol{x}} U) \Vert _{L^2_e(Q_T)} + \Vert c \nabla _{\boldsymbol{x}} \varPi ^{\nabla }_{h_{\boldsymbol{x}}}(\varPi _{h_t}^{\partial _t}- {{\,\textrm{Id}\,}}) V \Vert _{L^2_e(Q_T)}\bigr ), \end{aligned}$$

where $\beta := 2\sqrt{C_\varOmega ^2/c_0^2 + 4T^2}$ is the reciprocal of the inf–sup constant in Proposition 4.

##### Proof

We use Proposition 4 and Galerkin orthogonality to first obtain, for any $(W_{\boldsymbol{h}},Z_{\boldsymbol{h}}) \in (Q_{\boldsymbol{h}}(Q_T))^2$,

36 $$\begin{aligned} \begin{aligned}&\Vert (W_{\boldsymbol{h}}-U_{\boldsymbol{h}},Z_{\boldsymbol{h}}-V_{\boldsymbol{h}})\Vert _{\mathcal {V}_{e,\boldsymbol{h}}(Q_T)} \\  &\quad \quad \le \beta \sup _{0\ne (\lambda _{\boldsymbol{h}},\chi _{\boldsymbol{h}}) \in (\partial _t Q_{\boldsymbol{h}}(Q_T))^2} \frac{\mathcal {A}((W_{\boldsymbol{h}}- U_{\boldsymbol{h}},Z_{\boldsymbol{h}} - V_{\boldsymbol{h}}),(\lambda _{\boldsymbol{h}},\chi _{\boldsymbol{h}}))}{\Vert (\lambda _{\boldsymbol{h}},\chi _{\boldsymbol{h}}) \Vert _{\mathcal {W}_{e,\boldsymbol{h}}(Q_T)}} \\  &\quad \quad = \beta \sup _{0\ne (\lambda _{\boldsymbol{h}},\chi _{\boldsymbol{h}}) \in (\partial _t Q_{\boldsymbol{h}}(Q_T))^2} \frac{\mathcal {A}((W_{\boldsymbol{h}}- U,Z_{\boldsymbol{h}} - V),(\lambda _{\boldsymbol{h}},\chi _{\boldsymbol{h}}))}{\Vert (\lambda _{\boldsymbol{h}},\chi _{\boldsymbol{h}}) \Vert _{\mathcal {W}_{e,\boldsymbol{h}}(Q_T)}} \end{aligned} \end{aligned}$$

where $\beta = 2\sqrt{C_\varOmega ^2/c_0^2 + 4T^2}$. Using the definition of $\mathcal {A}$ in (11), we write

37 $$\begin{aligned} \begin{aligned}&\mathcal {A}((W_{\boldsymbol{h}}- U, Z_{\boldsymbol{h}} - V),(\lambda _{\boldsymbol{h}},\chi _{\boldsymbol{h}})) \\  &\quad = (\partial _t (Z_{\boldsymbol{h}} - V), \lambda _{\boldsymbol{h}})_{L^2_e(Q_T)} + (c^2\nabla _{\boldsymbol{x}} (W_{\boldsymbol{h}}-U), \nabla _{\boldsymbol{x}} \lambda _{\boldsymbol{h}})_{L^2_e(Q_T)} \\  &\quad \quad - (\partial _t (W_{\boldsymbol{h}}-U), \chi _{\boldsymbol{h}})_{L^2_e(Q_T)} + ((Z_{\boldsymbol{h}}-V), \chi _{\boldsymbol{h}})_{L^2_e(Q_T)} \\  &\quad =: I_1 + I_2 + I_3 + I_4. \end{aligned} \end{aligned}$$

Now, we take $W_{\boldsymbol{h}} = \varPi ^\nabla _{h_{\boldsymbol{x}}} \varPi _{h_t}^{\partial _t}U$ and $Z_{\boldsymbol{h}} = \varPi ^\nabla _{h_{\boldsymbol{x}}} \varPi _{h_t}^{\partial _t}V$. Then, using the commutativity properties in Lemma 4, together with (30), we obtain

$$\begin{aligned} \begin{aligned} I_1&= (\partial _t (\varPi ^{\nabla }_{h_{\boldsymbol{x}}}\varPi _{h_t}^{\partial _t}V - V), \lambda _{\boldsymbol{h}})_{L^2_e(Q_T)} = (\partial _t (\varPi _{h_t}^{\partial _t}\varPi ^{\nabla }_{h_{\boldsymbol{x}}}V - V), \lambda _{\boldsymbol{h}})_{L^2_e(Q_T)} \\  &= (\partial _t ( \varPi ^{\nabla }_{h_{\boldsymbol{x}}}V - V), \lambda _{\boldsymbol{h}})_{L^2_e(Q_T)} = ((\varPi ^{\nabla }_{h_{\boldsymbol{x}}}- {{\,\textrm{Id}\,}}) \partial _t V), \lambda _{\boldsymbol{h}})_{L^2_e(Q_T)}, \end{aligned} \end{aligned}$$

from which, using the Cauchy-Schwarz inequality, we obtain

38 $$\begin{aligned} \begin{aligned} I_1&\le \Vert (\varPi ^{\nabla }_{h_{\boldsymbol{x}}}- \text {Id}) \partial _t V \Vert _{L^2_e(Q_T)} \Vert (\lambda _{\boldsymbol{h}},\chi _{\boldsymbol{h}})\Vert _{\mathcal {W}_{e,\boldsymbol{h}}(Q_T)}. \end{aligned} \end{aligned}$$

Moreover, with (29) and integration by parts, where the regularity assumption in (35) is used, we deduce

$$\begin{aligned} I_2&= (c^2 \nabla _{\boldsymbol{x}} (\varPi ^\nabla _{h_{\boldsymbol{x}}} \varPi _{h_t}^{\partial _t}U - U), \nabla _{\boldsymbol{x}} \lambda _{\boldsymbol{h}})_{L^2_e(Q_T)} \\  &= (c^2 \nabla _{\boldsymbol{x}} (\varPi _{h_t}^{\partial _t}U - U), \nabla _{\boldsymbol{x}} \lambda _{\boldsymbol{h}})_{L^2_e(Q_T)} \\  &= - (\nabla _{\boldsymbol{x}} \cdot (c^2 \nabla _{\boldsymbol{x}} (\varPi _{h_t}^{\partial _t}U - U)),\lambda _{\boldsymbol{h}})_{L^2_e(Q_T)} \\  &= - ((\varPi _{h_t}^{\partial _t}- \text {Id})\nabla _{\boldsymbol{x}} \cdot (c^2 \nabla _{\boldsymbol{x}} U), \lambda _{\boldsymbol{h}})_{L^2_e(Q_T)}, \end{aligned}$$

where, in the last step, we used the commutativity properties in Lemma 4. Using again the Cauchy-Schwarz inequality, we obtain

39 $$\begin{aligned} \begin{aligned} I_2 \le \Vert (\varPi _{h_t}^{\partial _t}- \text {Id}) \nabla _{\boldsymbol{x}} \cdot (c^2 \nabla _{\boldsymbol{x}} U) \Vert _{L^2_e(Q_T)} \Vert (\lambda _{\boldsymbol{h}},\chi _{\boldsymbol{h}})\Vert _{\mathcal {W}_{e,\boldsymbol{h}}(Q_T)}. \end{aligned} \end{aligned}$$

For $I_3$, starting as for $I_1$ and using that $(\partial _t U,\chi _{\boldsymbol{h}})_{L^2_e(Q_T)} = (V,\chi _{\boldsymbol{h}})_{L^2_e(Q_T)}$, we have

$$\begin{aligned} \begin{aligned} I_3 = - ((\varPi ^{\nabla }_{h_{\boldsymbol{x}}}- {{\,\textrm{Id}\,}}) \partial _t U, \chi _{\boldsymbol{h}})_{L^2_e(Q_T)} = - ((\varPi ^{\nabla }_{h_{\boldsymbol{x}}}- {{\,\textrm{Id}\,}}) V, \chi _{\boldsymbol{h}})_{L^2_e(Q_T)}. \end{aligned} \end{aligned}$$

We proceed by estimating $I_3 + I_4$ jointly, which allows to take advantage of the cancellation of the terms $(V, \chi _{\boldsymbol{h}})_{L^2_e(Q_T)}$. Using the definition (26) of $\mathcal {N}^\varOmega _{e,\boldsymbol{h}}$, together with the Cauchy-Schwarz inequality, gives

$$\begin{aligned} I_3 + I_4&= (\varPi ^{\nabla }_{h_{\boldsymbol{x}}}(\varPi _{h_t}^{\partial _t}- {{\,\textrm{Id}\,}}) V, \chi _{\boldsymbol{h}})_{L^2_e(Q_T)} \\  &= (c^2 \nabla _{\boldsymbol{x}} \varPi ^{\nabla }_{h_{\boldsymbol{x}}}(\varPi _{h_t}^{\partial _t}- {{\,\textrm{Id}\,}}) V, \nabla _{\boldsymbol{x}} \mathcal {N}^\varOmega _{e,\boldsymbol{h}}\chi _{\boldsymbol{h}})_{L^2_e(Q_T)} \\  &\le \Vert c \nabla _{\boldsymbol{x}} \varPi ^{\nabla }_{h_{\boldsymbol{x}}}(\varPi _{h_t}^{\partial _t}- {{\,\textrm{Id}\,}}) V \Vert _{L^2_e(Q_T)} \Vert \chi _{\boldsymbol{h}} \Vert _{\mathcal {N}^\varOmega _{e,\boldsymbol{h}}}, \end{aligned}$$

from which

40 $$\begin{aligned} I_3 + I_4 \le \Vert c \nabla _{\boldsymbol{x}} \varPi ^{\nabla }_{h_{\boldsymbol{x}}}(\varPi _{h_t}^{\partial _t}- {{\,\textrm{Id}\,}}) V \Vert _{L^2_e(Q_T)} \Vert (\lambda _{\boldsymbol{h}},\chi _{\boldsymbol{h}}) \Vert _{\mathcal {W}_{e,\boldsymbol{h}}(Q_T)}. \end{aligned}$$

By substituting the estimates (38)–(40) for $I_1$–$I_4$ into (37), and combining with (36), we obtain the desired claim. $\square$

#### Error Estimates in the $\mathcal {V}_{e,\boldsymbol{h}}(Q_T)$ Norm

In the following, the notation $Q_1 \lesssim Q_2$ (resp. $Q_1 \gtrsim Q_2$) means that $Q_1$ is bounded above (resp. below) by $\kappa \,Q_2$, where $\kappa >0$ is a constant independent of the mesh sizes $h_t$ and $h_{\boldsymbol{x}}$, as well as of the involved functions. We remark that, unlike in the previous sections, where the dependence on the final time *T* was explicitly stated, the hidden constants in the $\lesssim$ notation may also depend on *T*.

We adopt the notation, for  $s_t, s_{\boldsymbol{x}} \ge 1$,

$$\begin{aligned} \begin{aligned} H_{0,\bullet }^{s_t}(0,T)&:= H_{0,\bullet }^1(0,T) \cap H^{s_t}(0,T), \\ H_0^{s_{\boldsymbol{x}}}(\varOmega )&:= H_0^1(\varOmega ) \cap H^{s_{\boldsymbol{x}}}(\varOmega ). \end{aligned} \end{aligned}$$

We proceed by deriving explicit error estimates under the following assumptions on the discrete spaces.

##### Assumption 3

*(space discretization)* The finite dimensional space $S_{h_{\boldsymbol{x}}}(\varOmega ) \subset H_0^1(\varOmega )$ satisfies the following approximation property for some $p_{\boldsymbol{x}} \in \mathbb {N}$, $p_{\boldsymbol{x}} \ge 1$: there exists an operator $Q_{h_{\boldsymbol{x}}}^{p_{\boldsymbol{x}}}: H_0^1(\varOmega )\rightarrow S_{h_{\boldsymbol{x}}}(\varOmega )$ such that, for all $W\in H_0^{s_{\boldsymbol{x}}+1}(\varOmega )$ with $1 \le s_{\boldsymbol{x}} \le p_{\boldsymbol{x}}$, it satisfies

$$\begin{aligned} \Vert \nabla _{\boldsymbol{x}} (Q_{h_{\boldsymbol{x}}}^{p_{\boldsymbol{x}}} W - W) \Vert _{L^2(\varOmega )}&\lesssim h_{\boldsymbol{x}}^{s_{\boldsymbol{x}}} \Vert W \Vert _{H^{s_{\boldsymbol{x}}+1}(\varOmega )}. \end{aligned}$$

##### Assumption 4

*(time discretization)* The finite dimensional space $S_{h_t}(0,T) \subset H_{0,\bullet }^1(0,T)$ satisfies the following approximation property for some $p_t \in \mathbb {N}$, $p_t \ge 1$: there exists an operator $Q_{h_t}^{p_t}: H_{0,\bullet }^1(0,T) \rightarrow S_{h_t}(0,T)$ such that, for all $w \in H_{0,\bullet }^{s_t+1}(0,T)$ with $1 \le s_t \le p_t$, it satisfies

$$\begin{aligned} \Vert \partial _t (Q_{h_t}^{p_t} w - w) \Vert _{L^2(0,T)}&\lesssim h_t^{s_t} \Vert \partial _t^{s_t+1} w \Vert _{L^2(0,T)}. \end{aligned}$$

##### Remark 10

In the case of discrete spaces consisting of continuous piecewise polynomials on shape-regular, simplicial meshes of $\varOmega$ and arbitrary meshes of (0, *T*), Assumptions 3 and 4 are both satisfied, e.g., with the quasi-interpolation operator defined in Section [10, §22.3]; see [10, Theorem 22.6]. For one-dimensional B-splines, Assumption 4 is satisfied, e.g., with the quasi-interpolant in [26, Theorem 18] (see also references therein). $\blacksquare$

Under Assumption 4, we have the following approximation result for the operator $\varPi _{h_t}^{\partial _t}$.

##### Lemma 6

For all $W \in H_{0,\bullet }^{s_t+1}(0,T;L^2(\varOmega ))$, with $1 \le s_t \le p_t$ and $p_t$ as in Assumption 4, it holds true that

$$\begin{aligned} \Vert ( \varPi _{h_t}^{\partial _t}-{{\,\textrm{Id}\,}}) W \Vert _{L^2_e(Q_T)} \lesssim h^{s_t+1}_t \Vert \partial _t^{s_t+1} W \Vert _{L_e^2(Q_T)}. \end{aligned}$$

##### Proof

We note that

41 $$\begin{aligned} \Vert ( \varPi _{h_t}^{\partial _t}-{{\,\textrm{Id}\,}})W \Vert _{L^2_e(Q_T)}^2 = \int _\varOmega \Vert (\varPi _{h_t}^{\partial _t}-{{\,\textrm{Id}\,}}) W(\boldsymbol{x},\cdot )\Vert ^2_{L^2_e(0,T)} \textrm{d}\boldsymbol{x}. \end{aligned}$$

We can apply the *Aubin–Nitsche trick* to derive the following estimate:

42 $$\begin{aligned} \Vert (\varPi _{h_t}^{\partial _t}-{{\,\textrm{Id}\,}}) w\Vert _{L^2_e(0,T)} \lesssim h_t \Vert \partial _t(\varPi _{h_t}^{\partial _t}-{{\,\textrm{Id}\,}})w \Vert _{L^2_e(0,T)} \,\,\, \text {for all~} w\in H_{0,\bullet }^1(0,T), \end{aligned}$$

where we used the notation $\varPi _{h_t}^{\partial _t}$ to also denote the elliptic projector acting on functions in $H^1(0,T)$. Once we have proved (42), the result follows by inserting it within (41), and using Assumption 4.

To prove (42), we consider the dual problem: given $f \in L^2(0,T)$, find $z \in H^1_{0,\bullet }(0,T)$ such that

43 $$\begin{aligned} (\partial _t v, \partial _t z)_{L^2_e(0,T)} = (f, v)_{L^2_e(0,T)} \quad \text {for all~} v \in H^1_{0,\bullet }(0,T). \end{aligned}$$

By the Lax–Milgram lemma, this problem admits a unique solution. Moreover, using the Poincaré inequality and (9), we obtain

44 $$\begin{aligned} \begin{aligned} \Vert \partial _t z \Vert _{L^2_e(0,T)}^2 = (f,z)_{L^2_e(0,T)}&\le \Vert z \Vert _{L^2_e(0,T)} \Vert f \Vert _{L^2_e(0,T)} \\  &\lesssim \Vert \partial _t z\Vert _{L^2_e(0,T)} \Vert f \Vert _{L^2_e(0,T)}. \end{aligned} \end{aligned}$$

Moreover, the unique solution of (43) can be explicitly computed as the solution of

$$\begin{aligned} {\left\{ \begin{array}{ll} \partial _t^2 z(t) + T^{-1} \partial _t z(t) = f(t) &  t \in (0,T), \\ z(0) = 0, \quad \partial _t z(T) = 0, \end{array}\right. } \end{aligned}$$

from which, also using (44), it follows that

45 $$\begin{aligned} \Vert \partial _t^2 z \Vert _{L^2_e(0,T)} \lesssim \Vert \partial _t z \Vert _{L^2_e(0,T)} + \Vert f \Vert _{L^2_e(0,T)} \lesssim \Vert f\Vert _{L^2_e(0,T)}. \end{aligned}$$

Now, let us take $f = (\varPi _{h_t}^{\partial _t}-{{\,\textrm{Id}\,}})w$ in problem (43), and test the equation with the function $v = (\varPi _{h_t}^{\partial _t}-{{\,\textrm{Id}\,}})w$. Note that $(\varPi _{h_t}^{\partial _t}-{{\,\textrm{Id}\,}})w$ is an admissible right-hand side test function since $w\in H^1_{0,\bullet }(0,T)$. Then, using the definition of the elliptic projection $\varPi _{h_t}^{\partial _t}$, we obtain that, for every $z_{h_t} \in S_{h_t}(0,T)$,

$$\begin{aligned} \begin{aligned} \Vert (\varPi _{h_t}^{\partial _t}-{{\,\textrm{Id}\,}})w \Vert _{L^2_e(0,T)}^2&= (\partial _t (\varPi _{h_t}^{\partial _t}-{{\,\textrm{Id}\,}})w, \partial _t (z-z_{h_t}))_{L^2_e(0,T)}\\  &\le \Vert \partial _t (\varPi _{h_t}^{\partial _t}-{{\,\textrm{Id}\,}})w \Vert _{L^2_e(0,T)} \Vert \partial _t (z-z_{h_t}) \Vert _{L^2_e(0,T)}, \end{aligned} \end{aligned}$$

where in the last step we used the Cauchy–Schwarz inequality. Using Assumption 4 with $s_t=1$, (9), and (45), we get

$$\begin{aligned} \inf _{z_{h_t} \in S_{h_t}(0,T)} \Vert \partial _t (z-z_{h_t}) \Vert _{L^2_e(0,T)} \lesssim h_t \Vert \partial _t^2 z \Vert _{L^2_e(0,T)} \lesssim h_t \Vert (\varPi _{h_t}^{\partial _t}-{{\,\textrm{Id}\,}}) w \Vert _{L^2_e(0,T)}. \end{aligned}$$

Combining the above inequalities yields (42) and the proof is complete. $\square$

The following lemma establishes the approximation properties of compositions of the projectors needed to prove explicit convergence estimates.

##### Lemma 7

Let $1 \le s_{\boldsymbol{x}} \le p_{\boldsymbol{x}}$ and $1 \le s_t \le p_t$, with $p_{\boldsymbol{x}}$ and $p_t$ as in Assumptions 3 and 4, respectively. Then,

(i) for any $$V \in H^1(0,T;H_0^{s_{\boldsymbol{x}} +1}(\varOmega ))$$,
  $$\begin{aligned} \Vert (\varPi ^{\nabla }_{h_{\boldsymbol{x}}}-{{\,\textrm{Id}\,}}) \partial _t V \Vert _{{L^2_e(Q_T)}} \lesssim h_{\boldsymbol{x}}^{s_{\boldsymbol{x}}}\Vert \partial _t V \Vert _{L^2(0,T;H^{s_{\boldsymbol{x}}+1}(\varOmega ))}; \end{aligned}$$
(ii) for any *U* such that $\nabla _{\boldsymbol{x}} \cdot (c^2 \nabla _{\boldsymbol{x}} U) \in H_{0,\bullet }^{s_t+1}(0,T;L^2(\varOmega ))$,
  $$\begin{aligned} \Vert (\varPi _{h_t}^{\partial _t}- {{\,\textrm{Id}\,}}) \nabla _{\boldsymbol{x}} \cdot (c^2 \nabla _{\boldsymbol{x}} U) \Vert _{L^2_e(Q_T)} \lesssim h_t^{s_t+1}\Vert \partial _t^{s_t+1} \nabla _{\boldsymbol{x}} \cdot (c^2 \nabla _{\boldsymbol{x}} U) \Vert _{L^2(Q_T)}; \end{aligned}$$
(iii) for any $V \in H_{0,\bullet }^{s_t + 1}(0,T;H^1_0(\varOmega ))$,
  $$\begin{aligned} \Vert c \nabla _{\boldsymbol{x}} \varPi ^{\nabla }_{h_{\boldsymbol{x}}}(\varPi _{h_t}^{\partial _t}- {{\,\textrm{Id}\,}}) V \Vert _{L^2_e(Q_T)} \lesssim h_t^{s_t+1}\Vert \partial _t^{s_t+1} V\Vert _{L^2(0,T;H^1(\varOmega ))}; \end{aligned}$$
(iv) for any $U \in H_{0,\bullet }^{s_t+1}(0,T;L^2(\varOmega )) \cap H^1(0,T;H_0^{s_{\boldsymbol{x}}+1}(\varOmega ))$,
  $$\begin{aligned} \Vert \partial _t (\varPi ^{\nabla }_{h_{\boldsymbol{x}}} \varPi _{h_t}^{\partial _t}- {{\,\textrm{Id}\,}})U\Vert _{{L^2_e(Q_T)}}&\lesssim h_t^{s_t}\Vert \partial _t^{s_t+1} U\Vert _{L^2(Q_T)} \\  &\quad + h^{s_{\boldsymbol{x}}}_{\boldsymbol{x}} \Vert \partial _t U \Vert _{L^2(0,T;H^{s_{\boldsymbol{x}} + 1}(\varOmega ))}; \end{aligned}$$
(v) for any $$V \in H_{0,\bullet }^{s_t+1}(0,T;H_0^1(\varOmega )) \cap L^2(0,T;H_0^{s_{\boldsymbol{x}}+1}(\varOmega ))$$,
  $$\begin{aligned} \Vert (\varPi ^{\nabla }_{h_{\boldsymbol{x}}}\varPi _{h_t}^{\partial _t}- {{\,\textrm{Id}\,}}) V\Vert _{{L^2_e(Q_T)}}&\lesssim h_t^{s_t+1}\Vert \partial _t^{s_t+1} V \Vert _{L^2(0,T;H^1(\varOmega ))} \\  &\quad + h_{\boldsymbol{x}}^{s_{\boldsymbol{x}}}\Vert V \Vert _{L^2(0,T;H^{s_{\boldsymbol{x}} + 1}(\varOmega ))}; \end{aligned}$$
(vi) for any $U \in H_{0,\bullet }^{s_t+1}(0,T;H^1(\varOmega )) \cap L^2(0,T;H_0^{s_{\boldsymbol{x}}+1}(\varOmega ))$,
  $$\begin{aligned} \Vert c\nabla _{\boldsymbol{x}} (\varPi ^{\nabla }_{h_{\boldsymbol{x}}}\varPi _{h_t}^{\partial _t}- {{\,\textrm{Id}\,}})U\Vert _{{L^2_e(Q_T)}}&\lesssim h^{s_t+1}_t \Vert \partial _t^{s_t+1} U \Vert _{L^2(0,T;H^1(\varOmega ))} \\  &\quad + h_{\boldsymbol{x}}^{s_{\boldsymbol{x}}}\Vert U \Vert _{L^2(0,T;H^{s_{\boldsymbol{x}} + 1}(\varOmega ))}; \end{aligned}$$
(vii) for any $V \in H_{0,\bullet }^{s_t + 1}(0,T;L^2(\varOmega )) \cap H^1(0,T;H_0^{s_{\boldsymbol{x}} +1}(\varOmega ))$,
  $$\begin{aligned} \Vert \partial _t (\varPi ^{\nabla }_{h_{\boldsymbol{x}}}\varPi _{h_t}^{\partial _t}- {{\,\textrm{Id}\,}}) V \Vert _{\mathcal {N}^\varOmega _{e,{\boldsymbol{h}}}}&\lesssim h_t^{s_t}\Vert \partial _t^{s_t+1} V\Vert _{L^2(Q_T)} \\  &\quad + h_{\boldsymbol{x}}^{s_{\boldsymbol{x}}}\Vert \partial _t V \Vert _{L^2(0,T;H^{s_{\boldsymbol{x}}+1}(\varOmega ))}; \end{aligned}$$
(viii) for any
  $$\begin{aligned}&U \in H_{0,\bullet }^{s_t+1}(0,T;H^1(\varOmega )) \cap H^1(0,T;H_0^{s_{\boldsymbol{x}}+1}(\varOmega )), \\  &V \in H_{0,\bullet }^{s_t+1}(0,T;H_0^1(\varOmega )) \cap H^1(0,T;H_0^{s_{\boldsymbol{x}}+1}(\varOmega )), \end{aligned}$$
  it holds true that
  $$\begin{aligned} \begin{aligned}&\Vert ((\varPi ^{\nabla }_{h_{\boldsymbol{x}}}\varPi _{h_t}^{\partial _t}-{{\,\textrm{Id}\,}}) U,(\varPi ^{\nabla }_{h_{\boldsymbol{x}}}\varPi _{h_t}^{\partial _t}-{{\,\textrm{Id}\,}}) V) \Vert _{\mathcal {V}_{e,{\boldsymbol{h}}}(Q_T)} \\&\quad \quad \quad \lesssim h_t^{s_t}\Vert \partial _t^{s_t+1} U\Vert _{L^2(0,T;H^1(\varOmega ))}+ h^{s_{\boldsymbol{x}}}_{\boldsymbol{x}} \Vert \partial _t U \Vert _{L^2(0,T;H^{s_{\boldsymbol{x}} + 1}(\varOmega ))} \\  &\quad \quad \quad \quad + h_t^{s_t}\Vert \partial _t^{s_t+1} V\Vert _{L^2(0,T;H^1(\varOmega ))} + h_{\boldsymbol{x}}^{s_{\boldsymbol{x}}}\Vert \partial _t V \Vert _{L^2(0,T;H^{s_{\boldsymbol{x}}+1}(\varOmega ))}. \end{aligned} \end{aligned}$$

##### Proof

For the estimate in *(i)*, we apply the Poincaré inequality in space

$$\begin{aligned} \Vert (\varPi ^{\nabla }_{h_x} - {{\,\textrm{Id}\,}})\partial _tV \Vert _{L^2_e(Q_T)} \lesssim \Vert c \nabla _x(\varPi ^{\nabla }_{h_x} - {{\,\textrm{Id}\,}})\partial _tV\Vert _{L^2_e(Q_T)}, \end{aligned}$$

the approximation property in (31), and Assumption 3:

$$\begin{aligned} \begin{aligned}&\Vert c \nabla _x(\varPi ^{\nabla }_{h_x} - {{\,\textrm{Id}\,}})\partial _tV\Vert _{L_e^2(Q_T)} \\  &\quad \lesssim \inf _{W_{h_{\boldsymbol{x}}} \in S_{h_{\boldsymbol{x}}}(\varOmega ) \otimes L^2(0,T)} \Vert c\nabla _{\boldsymbol{x}} (W_{h_{\boldsymbol{x}}} - \partial _t V) \Vert _{L_e^2(Q_T)} \\  &\quad \lesssim h_{\boldsymbol{x}}^{s_{\boldsymbol{x}}} \left( \int _0^T \Vert \partial _t V(\cdot ,s) \Vert ^2_{H^{s_{\boldsymbol{x}}+1}(\varOmega )} e^{-s/T} \, \textrm{d}s\right) ^{\frac{1}{2}} \\  &\quad \lesssim h_{\boldsymbol{x}}^{s_{\boldsymbol{x}}} \Vert \partial _t V \Vert _{L^2(0,T;H^{s_{\boldsymbol{x}}+1}(\varOmega ))}. \end{aligned} \end{aligned}$$

To obtain *(ii)*, we simply use Lemma 6, while *(iii)* is obtained by combining the stability property in (33) with Lemma 6.

In the subsequent steps of the proof, we repeatedly use the following identities, which follow from Lemma 4:

$$\begin{aligned} \varPi ^{\nabla }_{h_{\boldsymbol{x}}}\varPi _{h_t}^{\partial _t}- {{\,\textrm{Id}\,}}&= \varPi _{h_t}^{\partial _t}\varPi ^{\nabla }_{h_{\boldsymbol{x}}}- {{\,\textrm{Id}\,}}\\  &= \varPi _{h_t}^{\partial _t}- {{\,\textrm{Id}\,}}+ \varPi _{h_t}^{\partial _t}(\varPi ^{\nabla }_{h_{\boldsymbol{x}}} - {{\,\textrm{Id}\,}}) \\  &= \varPi ^{\nabla }_{h_{\boldsymbol{x}}}(\varPi _{h_t}^{\partial _t}- {{\,\textrm{Id}\,}}) + \varPi ^{\nabla }_{h_{\boldsymbol{x}}} - {{\,\textrm{Id}\,}}. \end{aligned}$$

To prove the estimate in *(iv)*, we compute

$$\begin{aligned}&\Vert \partial _t(\varPi ^{\nabla }_{h_{\boldsymbol{x}}}\varPi _{h_t}^{\partial _t}- {{\,\textrm{Id}\,}})U\Vert _{{L^2_e(Q_T)}} \\  &\quad \le \Vert \partial _t (\varPi _{h_t}^{\partial _t}- {{\,\textrm{Id}\,}})U \Vert _{{L^2_e(Q_T)}} + \Vert \partial _t \varPi _{h_t}^{\partial _t}(\varPi ^{\nabla }_{h_{\boldsymbol{x}}}- {{\,\textrm{Id}\,}})U \Vert _{{L^2_e(Q_T)}} \\&\quad \le \Vert \partial _t (\varPi _{h_t}^{\partial _t}- {{\,\textrm{Id}\,}}) U \Vert _{{L^2_e(Q_T)}} + \Vert (\varPi ^{\nabla }_{h_{\boldsymbol{x}}}- {{\,\textrm{Id}\,}}) \partial _t U \Vert _{{L^2_e(Q_T)}}, \\  &\quad \lesssim \Vert \partial _t (\varPi _{h_t}^{\partial _t}- {{\,\textrm{Id}\,}}) U \Vert _{{L^2_e(Q_T)}} + \Vert c \nabla _{\boldsymbol{x}} (\varPi ^{\nabla }_{h_{\boldsymbol{x}}}- {{\,\textrm{Id}\,}}) \partial _t U \Vert _{{L^2_e(Q_T)}}, \end{aligned}$$

where we also used the stability property in (34), the Poincaré inequality in space and the commutativity properties in Lemma 4. Then, *(iv)* follows from the approximation properties (31) and (32), combined with Assumptions 3 and 4.

For the inequality in *(v)*, the Poincaré inequality in space, the stability property in (33), and the commutativity properties in Lemma 4 imply

$$\begin{aligned}&\Vert (\varPi ^{\nabla }_{h_{\boldsymbol{x}}}\varPi _{h_t}^{\partial _t}- {{\,\textrm{Id}\,}}) V \Vert _{{L^2_e(Q_T)}} \\  &\quad \le \Vert \varPi ^{\nabla }_{h_{\boldsymbol{x}}}(\varPi _{h_t}^{\partial _t}- {{\,\textrm{Id}\,}}) V\Vert _{{L^2_e(Q_T)}} + \Vert (\varPi ^{\nabla }_{h_{\boldsymbol{x}}}-{{\,\textrm{Id}\,}}) V\Vert _{{L^2_e(Q_T)}} \\  &\quad \lesssim \Vert \nabla _{\boldsymbol{x}} \varPi ^{\nabla }_{h_{\boldsymbol{x}}}(\varPi _{h_t}^{\partial _t}- {{\,\textrm{Id}\,}}) V\Vert _{{L^2_e(Q_T)}} + \Vert \nabla _{\boldsymbol{x}} (\varPi ^{\nabla }_{h_{\boldsymbol{x}}}-{{\,\textrm{Id}\,}}) V\Vert _{{L^2_e(Q_T)}} \\  &\quad \lesssim \Vert (\varPi _{h_t}^{\partial _t}- {{\,\textrm{Id}\,}}) \nabla _{\boldsymbol{x}} V\Vert _{{L^2_e(Q_T)}} + \Vert \nabla _{\boldsymbol{x}} (\varPi ^{\nabla }_{h_{\boldsymbol{x}}}- {{\,\textrm{Id}\,}}) V\Vert _{{L^2_e(Q_T)}}. \end{aligned}$$

We conclude with Lemma 6 and the approximation property (31) of $\varPi ^{\nabla }_{h_{\boldsymbol{x}}}$, combined with Assumption 3.

For *(vi)*, we proceed similarly, and compute, with the stability property in (33) and the commutativity properties in Lemma 4

$$\begin{aligned}&\Vert c \nabla _{\boldsymbol{x}} (\varPi ^{\nabla }_{h_{\boldsymbol{x}}}\varPi _{h_t}^{\partial _t}- {{\,\textrm{Id}\,}})U\Vert _{{L^2_e(Q_T)}} \\  &\quad \le \Vert c \nabla _{\boldsymbol{x}} \varPi ^{\nabla }_{h_{\boldsymbol{x}}}(\varPi _{h_t}^{\partial _t}- {{\,\textrm{Id}\,}})U \Vert _{{L^2_e(Q_T)}} + \Vert c \nabla _{\boldsymbol{x}} (\varPi ^{\nabla }_{h_{\boldsymbol{x}}}- {{\,\textrm{Id}\,}})U \Vert _{{L^2_e(Q_T)}} \\  &\quad \le \Vert (\varPi _{h_t}^{\partial _t}- {{\,\textrm{Id}\,}}) c\nabla _{\boldsymbol{x}} U \Vert _{{L^2_e(Q_T)}}+ \Vert c \nabla _{\boldsymbol{x}} (\varPi ^{\nabla }_{h_{\boldsymbol{x}}}- {{\,\textrm{Id}\,}})U \Vert _{{L^2_e(Q_T)}}, \end{aligned}$$

and conclude exactly as for *(v)*.

For *(vii)*, we use Lemma 3 to first obtain

$$\begin{aligned} \Vert \partial _t (\varPi ^{\nabla }_{h_{\boldsymbol{x}}}\varPi _{h_t}^{\partial _t}- {{\,\textrm{Id}\,}}) V\Vert _{\mathcal {N}^\varOmega _{e,{\boldsymbol{h}}}}&\lesssim \Vert \partial _t (\varPi ^{\nabla }_{h_{\boldsymbol{x}}}\varPi _{h_t}^{\partial _t}- {{\,\textrm{Id}\,}}) V\Vert _{{L^2_e(Q_T)}}, \end{aligned}$$

and then proceed as in *(iv)*.

Finally, the projection error estimate in the $\mathcal {V}_{e,\boldsymbol{h}}(Q_T)$ norm stated in *(viii)* readily follows from *(iv)*–*(vii)* and the Poincaré inequality applied in space and in time. $\square$

##### Remark 11

Under a standard elliptic regularity assumption in space, improved estimates for the projection error associated with $\varPi ^{\nabla }_{h_{\boldsymbol{x}}}$ in the $L^2_e(Q_T)$ norm can be derived using a duality argument. We emphasize that this assumption is not needed for the error estimate in Theorem 5. However, in Section 4.2.3 below, we rely on it, along with the resulting improved projection error estimates, to derive error bounds in the $L^2$ norm. $\blacksquare$

##### Remark 12

When *U* and *V* are sufficiently smooth, we deduce from Lemma 5 and *(i)*–*(iii)* in Lemma 7 that

$$\begin{aligned} \Vert (\varPi ^{\nabla }_{h_{\boldsymbol{x}}}\varPi _{h_t}^{\partial _t}U- U_{\boldsymbol{h}}, \varPi ^{\nabla }_{h_{\boldsymbol{x}}}\varPi _{h_t}^{\partial _t}V - V_{\boldsymbol{h}})\Vert _{\mathcal {V}_{e,\boldsymbol{h}}(Q_T)} \lesssim C(U,V) \left( h_{\boldsymbol{x}}^{s_{\boldsymbol{x}}}+h_t^{s_t+1}\right) , \end{aligned}$$

where we have absorbed the dependence on the analytical solution (*U*, *V*) and its derivatives into the positive constant *C*(*U*, *V*), while, for the projection error, the best achievable estimate in the norm $\Vert \cdot \Vert _{\mathcal {V}_{e,\boldsymbol{h}}(Q_T)}$ is

$$\begin{aligned} \Vert ((\varPi ^{\nabla }_{h_{\boldsymbol{x}}}\varPi _{h_t}^{\partial _t}-{{\,\textrm{Id}\,}}) U,(\varPi ^{\nabla }_{h_{\boldsymbol{x}}}\varPi _{h_t}^{\partial _t}-{{\,\textrm{Id}\,}}) V)\Vert _{\mathcal {V}_{e,\boldsymbol{h}}(Q_T)}\lesssim C(U,V) \left( h_{\boldsymbol{x}}^{s_{\boldsymbol{x}}}+h_t^{s_t}\right) . \end{aligned}$$

$$\blacksquare $$

Combining Lemmas 5 and 7 yields the main result.

##### Theorem 5

Let us assume the regularity on the data as in Assumption 1. Let (*U*, *V*) be the unique solution to (24), and $(U_{\boldsymbol{h}},V_{\boldsymbol{h}})$ be the unique discrete solution to (25). Let $1 \le s_{\boldsymbol{x}} \le p_{\boldsymbol{x}}$ and $1 \le s_t \le p_t$, with $p_{\boldsymbol{x}}$ and $p_t$ as in Assumptions 3 and 4, respectively. Assume that (*U*, *V*) satisfies

46 $$\begin{aligned} \begin{aligned}&U \in H_{0,\bullet }^{s_t+1}(0,T;H^1(\varOmega )) \cap H^1(0,T;H_0^{s_{\boldsymbol{x}}+1}(\varOmega )), \\  &V \in H_{0,\bullet }^{s_t+1}(0,T;H^1(\varOmega )) \cap H^1(0,T;H_0^{s_{\boldsymbol{x}}+1}(\varOmega )), \\  &\nabla _{\boldsymbol{x}} \cdot (c^2 \nabla _{\boldsymbol{x}} U) \in H_{0,\bullet }^{s_t+1}(0,T;L^2(\varOmega )). \end{aligned} \end{aligned}$$

Then, we have the following error estimate:

$$\begin{aligned} \Vert (U,V) -&(U_{\boldsymbol{h}},V_{\boldsymbol{h}})\Vert _{\mathcal {V}_{e,\boldsymbol{h}}(Q_T)} \\  &\lesssim h^{s_t}_t \Vert \partial _t^{s_t+1} U \Vert _{L^2(0,T;H^1(\varOmega ))} + h^{s_{\boldsymbol{x}}}_{\boldsymbol{x}} \Vert \partial _t U \Vert _{L^2(0,T;H^{s_{\boldsymbol{x}}+1}(\varOmega ))} \\&\quad + h_t^{s_t}\Vert \partial _t^{s_t+1} V\Vert _{L^2(0,T;H^1(\varOmega ))} + h_{\boldsymbol{x}}^{s_{\boldsymbol{x}}}\Vert \partial _t V \Vert _{L^2(0,T;H^{s_{\boldsymbol{x}}+1}(\varOmega ))} \\  &\quad + h_t^{s_t+1} \Vert \partial _t^{s_t+1} \nabla _{\boldsymbol{x}} \cdot (c^2 \nabla _{\boldsymbol{x}} U) \Vert _{L^2(Q_T)}. \end{aligned}$$

Moreover, for the term $\Vert c \nabla _{\boldsymbol{x}} (U - U_{\boldsymbol{h}}) \Vert _{L^2_e(Q_T)}$ alone, we have

$$\begin{aligned} \Vert c \nabla _{\boldsymbol{x}}&(U - U_{\boldsymbol{h}}) \Vert _{L^2_e(Q_T)} \\  &\lesssim h^{s_t+1}_t \Vert \partial _t^{s_t+1} U \Vert _{L^2(0,T;H^1(\varOmega ))} \\  &\quad + h_{\boldsymbol{x}}^{s_{\boldsymbol{x}}}\Vert U \Vert _{L^2(0,T;H^{s_{\boldsymbol{x}} + 1}(\varOmega ))} +h_{\boldsymbol{x}}^{s_{\boldsymbol{x}}}\Vert \partial _t V \Vert _{L^2(0,T;H^{s_{\boldsymbol{x}} + 1}(\varOmega ))} \\  &\quad + h_t^{s_t+1} (\Vert \partial _t^{s_t+1} \nabla _{\boldsymbol{x}} \cdot (c^2 \nabla _{\boldsymbol{x}} U) \Vert _{L^2(Q_T)} + \Vert \partial _t^{s_t+1} V\Vert _{L^2(0,T;H^1(\varOmega ))}). \end{aligned}$$

##### Proof

For all $(W_{\boldsymbol{h}},Z_{\boldsymbol{h}}) \in (Q_{\boldsymbol{h}}(Q_T))^2$, with the triangle inequality, we deduce

47 $$\begin{aligned} \begin{aligned} \Vert (U,V) - (U_{\boldsymbol{h}},V_{\boldsymbol{h}})\Vert _{\mathcal {V}_{e,\boldsymbol{h}}(Q_T)}&\le \Vert (U,V) - (W_{\boldsymbol{h}},Z_{\boldsymbol{h}})\Vert _{\mathcal {V}_{e,\boldsymbol{h}}(Q_T)} \\  &\quad + \Vert (W_{\boldsymbol{h}},Z_{\boldsymbol{h}}) - (U_{\boldsymbol{h}},V_{\boldsymbol{h}})\Vert _{\mathcal {V}_{e,\boldsymbol{h}}(Q_T)}. \end{aligned} \end{aligned}$$

We choose $W_{\boldsymbol{h}}:= \varPi ^{\nabla }_{h_{\boldsymbol{x}}}\varPi _{h_t}^{\partial _t}U$ and $Z_{\boldsymbol{h}}:= \varPi ^{\nabla }_{h_{\boldsymbol{x}}}\varPi _{h_t}^{\partial _t}V$  where the projectors are defined in (29) and (30). Then, we obtain the estimate in the $\Vert \cdot \Vert _{\mathcal {V}_{e,\boldsymbol{h}}(Q_T)}$ norm by using *(viii)* in Lemma 7 to estimate $\Vert (U,V) - (W_{\boldsymbol{h}},Z_{\boldsymbol{h}})\Vert _{\mathcal {V}_{e,\boldsymbol{h}}(Q_T)}$, and combining Lemma 5 with *(i)*–*(iii)* in Lemma 7 to estimate $\Vert (W_{\boldsymbol{h}},Z_{\boldsymbol{h}}) - (U_{\boldsymbol{h}},V_{\boldsymbol{h}})\Vert _{\mathcal {V}_{e,\boldsymbol{h}}(Q_T)}$.

For the second estimate, the triangle inequality, together with the definition (28) of $\Vert \cdot \Vert _{\mathcal {V}_{e,\boldsymbol{h}}(Q_T)}$, gives

48 $$\begin{aligned}&\nonumber \Vert c \nabla _{\boldsymbol{x}} (U - U_{\boldsymbol{h}}) \Vert _{L^2_e(Q_T)} \\  &\quad \le \Vert c \nabla _{\boldsymbol{x}} (W_{\boldsymbol{h}} - U_{\boldsymbol{h}}) \Vert _{L^2_e(Q_T)} + \Vert c \nabla _{\boldsymbol{x}} (W_{\boldsymbol{h}}-U) \Vert _{L^2_e(Q_T)} \nonumber \\  &\quad \le \Vert (W_{\boldsymbol{h}},Z_{\boldsymbol{h}}) - (U_{\boldsymbol{h}},V_{\boldsymbol{h}})\Vert _{\mathcal {V}_{e,\boldsymbol{h}}(Q_T)} + \Vert c \nabla _{\boldsymbol{x}} (W_{\boldsymbol{h}}-U) \Vert _{L^2_e(Q_T)}. \end{aligned}$$

We choose again $W_{\boldsymbol{h}}:= \varPi ^{\nabla }_{h_{\boldsymbol{x}}}\varPi _{h_t}^{\partial _t}U$ and $Z_{\boldsymbol{h}}:= \varPi ^{\nabla }_{h_{\boldsymbol{x}}}\varPi _{h_t}^{\partial _t}V$, and conclude estimating $\Vert (W_{\boldsymbol{h}},Z_{\boldsymbol{h}})-(U_{\boldsymbol{h}},V_{\boldsymbol{h}})\Vert _{\mathcal {V}_{e,\boldsymbol{h}}(Q_T)}$ as in the first part, and $\Vert c \nabla _{\boldsymbol{x}} (W_{\boldsymbol{h}}-U) \Vert _{L^2_e(Q_T)}$ by using *(vi)* in Lemma 7. $\square$

##### Remark 13

As pointed out in Remark 12, the projection error term dominates in (47), yielding

$$\begin{aligned} \Vert (U,V) - (U_{\boldsymbol{h}},V_{\boldsymbol{h}})\Vert _{\mathcal {V}_{e,\boldsymbol{h}}(Q_T)} \lesssim C(U,V)\left( h_{\boldsymbol{x}}^{s_{\boldsymbol{x}}}+h_t^{s_t}\right) , \end{aligned}$$

where, as in Remark 12, we have absorbed the dependence on the analytical solution (*U*, *V*) and its derivatives into the positive constant *C*(*U*, *V*). On the contrary, both terms on the right-hand side of (48) admit similar bounds, allowing to obtain

$$\begin{aligned} \Vert c \nabla _{\boldsymbol{x}} (U - U_{\boldsymbol{h}}) \Vert _{L^2_e(Q_T)} \lesssim C(U,V) \left( h_{\boldsymbol{x}}^{s_{\boldsymbol{x}}}+h_t^{s_t+1}\right) . \end{aligned}$$

Furthermore, due to the norm equivalence (10) in Remark 2, we readily have

$$\begin{aligned} \Vert c \nabla _{\boldsymbol{x}} (U - U_{\boldsymbol{h}}) \Vert _{L^2(Q_T)} \lesssim C(U,V) \left( h_{\boldsymbol{x}}^{s_{\boldsymbol{x}}}+h_t^{s_t+1}\right) . \end{aligned}$$

Also estimates with the same rates for $\Vert \partial _t (U - U_{\boldsymbol{h}})\Vert _{L^2(Q_T)}$, $\Vert U - U_{\boldsymbol{h}}\Vert _{L^2(Q_T)}$ and $\Vert V - V_{\boldsymbol{h}}\Vert _{L^2(Q_T)}$ immediately follow. However, in the following section, we derive estimates with improved rates for the errors $U- U_{\boldsymbol{h}}$ and $V-V_{\boldsymbol{h}}$ in the $L^2$ norm (see Theorem 7 and Remark 16). $\blacksquare$

##### Remark 14

We recall from [11, Theorem 6, §7.2.3, Pag. 412] the following result. For smooth $\partial \varOmega$, assume that

$$\begin{aligned} \begin{aligned} U_0 \in H^{m+1}(\varOmega ), \quad V_0 \in H^m(\varOmega ), \quad F \in H^k(0,T;H^{m-k}(\varOmega )) \quad \text {for~}k = 0,\ldots ,m, \end{aligned} \end{aligned}$$

and that the following *m*-th compatibility conditions hold: for $\{u_k\}_{k=0}^m$ defined recursively by

$$\begin{aligned} {\left\{ \begin{array}{ll} u_0 := U_0, \quad u_1 := V_0, &  \\ u_k := \partial _t^{k-2}F(\cdot ,0) + \nabla _{\boldsymbol{x}}\cdot (c^2 \nabla _{\boldsymbol{x}} u_{k-2}) &  \quad \text {for } k=2,\dots ,m, \end{array}\right. } \end{aligned}$$

we require that

$$\begin{aligned} u_k \in H_0^1(\varOmega ) \quad \text {for } k=0,\ldots ,m. \end{aligned}$$

Then, the solution *U* of (1) satisfies

$$\begin{aligned} U \in H^k(0,T;H^{m+1-k}(\varOmega )) \quad \text {for } k=0,\ldots ,m+1. \end{aligned}$$

For $V=\partial _t U$, this implies

$$\begin{aligned} V \in H^k(0,T;H^{m-k}(\varOmega )) \quad \text {for } k=0,\ldots ,m. \end{aligned}$$

Consequently, the regularity assumptions in Theorem 5 are ensured by suitable assumptions on the regularity of the wave velocity, the initial data, and the source term, together with compatibility conditions of sufficiently high order. Moreover, assumption (35) in Lemma 5, is guaranteed, for instance, if $c \in W^{1,\infty }(\varOmega )$, $U_0 \in H^3(\varOmega )$, $V_0 \in H^2(\varOmega )$, $F \in H^k(0,T;H^{2-k}(\varOmega ))$ for $k=0,1,2$, and $F(\cdot ,0) + \nabla _{\boldsymbol{x}} \cdot (c^2 \nabla _{\boldsymbol{x}} U_0) \in H_0^1(\varOmega )$. $\blacksquare$

#### Error Estimates in the $L^2$ Norm

In this section, we derive improved error estimates for $\Vert U - U_{\boldsymbol{h}}\Vert _{L_e^2(Q_T)}$ and $\Vert V- V_{\boldsymbol{h}}\Vert _{L_e^2(Q_T)}$ under the following standard elliptic regularity assumption in space.

##### Assumption 6

*(elliptic regularity)* We assume that, for any $g \in L^2(\varOmega )$, the unique solution to the problem: find $\varphi \in H_0^1(\varOmega )$ such that

$$\begin{aligned} (c^2 \nabla _{\boldsymbol{x}} \varphi , \nabla _{\boldsymbol{x}} \psi )_{L^2(\varOmega )} = (g,\psi )_{L^2(\varOmega ) \quad } \quad \text {for all~} \psi \in H^1_0(\varOmega ) \end{aligned}$$

belongs to $H^2(\varOmega )$ and satisfies the stability estimate $\Vert \varphi \Vert _{H^2(\varOmega )} \lesssim \Vert g \Vert _{L^2(\varOmega )}$.

##### Remark 15

Assumption 6 is satisfied when $c^2$ is a Lipschitz function in $\varOmega$, and $\varOmega$ is convex and Lipschitz, or $\partial \varOmega$ is of class $C^{1,1}$. $\blacksquare$

Under Assumptions 3 and 6, a standard duality argument yields

49 $$\begin{aligned} \Vert (\varPi ^{\nabla }_{h_{\boldsymbol{x}}}-{{\,\textrm{Id}\,}})W\Vert _{L^2_e(Q_T)}\lesssim h_{\boldsymbol{x}}\Vert \nabla _{\boldsymbol{x}}(\varPi ^{\nabla }_{h_{\boldsymbol{x}}}-{{\,\textrm{Id}\,}})W\Vert _{L^2_e(Q_T)} \end{aligned}$$

for all $W\in L^2(0,T;H^1_0(\varOmega ))$, which leads to error estimates in the $L^2_e(Q_T)$ norm with improved converge rates in $h_{\boldsymbol{x}}$. To achieve this, we first refine the projection error estimates *(i)* and *(v)* of Lemma 7.

##### Lemma 8

Let $1 \le s_{\boldsymbol{x}} \le p_{\boldsymbol{x}}$ and $1 \le s_t \le p_t$ , with $p_{\boldsymbol{x}}$ and $p_{t}$ as in Assumptions 3 and 4, respectively. Then, if also Assumption 6 is satisfied, we obtain

(i) for any $$W \in H^1(0,T;H_0^{s_{\boldsymbol{x}} +1}(\varOmega ))$$,
  $$\begin{aligned} \Vert (\varPi ^{\nabla }_{h_{\boldsymbol{x}}}-{{\,\textrm{Id}\,}}) \partial _t W \Vert _{{L^2_e(Q_T)}} \lesssim h_{\boldsymbol{x}}^{s_{\boldsymbol{x}}+1}\Vert \partial _t W \Vert _{L^2(0,T;H^{s_{\boldsymbol{x}}+1}(\varOmega ))}; \end{aligned}$$
(ii) for any $W \in H_{0,\bullet }^{s_t+1}(0,T;H_0^1(\varOmega )) \cap L^2(0,T;H_0^{s_{\boldsymbol{x}}+1}(\varOmega ))$,
  $$\begin{aligned} \Vert (\varPi ^{\nabla }_{h_{\boldsymbol{x}}}\varPi _{h_t}^{\partial _t}- {{\,\textrm{Id}\,}}) W\Vert _{{L^2_e(Q_T)}}&\lesssim h_t^{s_t+1}\Vert \partial _t^{s_t+1} W \Vert _{L^2(0,T;H^1(\varOmega ))} \\  &\quad + h_{\boldsymbol{x}}^{s_{\boldsymbol{x}}+1}\Vert W \Vert _{L^2(0,T;H^{s_{\boldsymbol{x}} + 1}(\varOmega ))}. \end{aligned}$$

##### Proof

The proofs of *(i)* and *(ii)* proceed analogously to those of *(i)* and *(v)* in Lemma 7, respectively, except that, instead of the Poincaré inequality in space, we use (49) to estimate terms of the form $\Vert (\varPi _{h_{\boldsymbol{x}}}^\nabla - {{\,\textrm{Id}\,}}) \cdot \Vert _{L^2_e(Q_T)}$, which gives the extra power of $h_{\boldsymbol{x}}$. $\square$

We now prove the main result of this section.

##### Theorem 7

In addition to the assumptions of Theorem 5, suppose that Assumption 6 is satisfied. Then, we have

50 $$\begin{aligned} \begin{aligned}&\Vert U - U_{\boldsymbol{h}}\Vert _{L_e^2(Q_T)} \\  &\quad \lesssim h^{s_t+1}_t \Vert \partial _t^{s_t+1} U \Vert _{L^2(0,T;H^1(\varOmega ))} + h^{s_{\boldsymbol{x}}+1}_{\boldsymbol{x}} \Vert U \Vert _{L^2(0,T;H^{s_{\boldsymbol{x}}+1}(\varOmega ))} \\  &\quad \quad + h_t^{s_t+1} (\Vert \partial _t^{s_t+1} \nabla _{\boldsymbol{x}} \cdot (c^2 \nabla _{\boldsymbol{x}} U) \Vert _{L^2(Q_T)} + \Vert \partial _t^{s_t+1} V\Vert _{L^2(0,T;H^1(\varOmega ))}), \\  &\quad \quad + h_{\boldsymbol{x}}^{s_{\boldsymbol{x}}+1} \Vert \partial _t V \Vert _{L^2(0,T;H^{s_{\boldsymbol{x}}+1}(\varOmega ))}, \end{aligned} \end{aligned}$$

51 $$\begin{aligned} \begin{aligned}&\Vert V - V_{\boldsymbol{h}}\Vert _{L_e^2(Q_T)} \\  &\quad \lesssim h^{s_t+1}_t \Vert \partial _t^{s_t+1} V \Vert _{L^2(0,T;H^1(\varOmega ))} + h^{s_{\boldsymbol{x}}+1}_{\boldsymbol{x}} \Vert \partial _t V \Vert _{L^2(0,T;H^{s_{\boldsymbol{x}}+1}(\varOmega ))} \\  &\quad \quad + h_t^{s_t+1} (\Vert \partial _t^{s_t+1} \nabla _{\boldsymbol{x}} \cdot (c^2 \nabla _{\boldsymbol{x}} U) \Vert _{L^2(Q_T)} + \Vert \partial _t^{s_t+1} V\Vert _{L^2(0,T;H^1(\varOmega ))}). \end{aligned} \end{aligned}$$

##### Proof

Using the triangle inequality, the Poincaré inequality in time, and the definition (28) of $\Vert \cdot \Vert _{\mathcal {V}_{e,\boldsymbol{h}}(Q_T)}$, we obtain

$$\begin{aligned} \Vert U - U_{\boldsymbol{h}}\Vert _{L_e^2(Q_T)}&\le \Vert \varPi ^{\nabla }_{h_{\boldsymbol{x}}}\varPi _{h_t}^{\partial _t}U - U_{\boldsymbol{h}} \Vert _{L_e^2(Q_T)} + \Vert (\varPi ^{\nabla }_{h_{\boldsymbol{x}}}\varPi _{h_t}^{\partial _t}- {{\,\textrm{Id}\,}}) U\Vert _{L_e^2(Q_T)} \\  &\hspace{-2cm} \le \Vert (\varPi ^{\nabla }_{h_{\boldsymbol{x}}}\varPi _{h_t}^{\partial _t}U- U_{\boldsymbol{h}}, \varPi ^{\nabla }_{h_{\boldsymbol{x}}}\varPi _{h_t}^{\partial _t}V - V_{\boldsymbol{h}})\Vert _{\mathcal {V}_{e,\boldsymbol{h}}(Q_T)} + \Vert (\varPi ^{\nabla }_{h_{\boldsymbol{x}}}\varPi _{h_t}^{\partial _t}- {{\,\textrm{Id}\,}}) U\Vert _{L_e^2(Q_T)}. \end{aligned}$$

We derive (50) combining Lemma 5, *(i)*, *(ii)* of Lemma 8, and *(ii)*, *(iii)* of Lemma 7. Inequality (51) is obtained similarly, except that, in the first step, the Poincaré inequality is not needed. We note that, in the derivation of (51), the term $h_{\boldsymbol{x}}^{s_{\boldsymbol{x}}+1}\Vert V \Vert _{L^2(0,T;H^{s_{\boldsymbol{x}} + 1}(\varOmega ))}$ arising in *(ii)* of Lemma 8 is estimated by a Poincaré inequality in time and then absorbed into the term $h^{s_{\boldsymbol{x}}+1}_{\boldsymbol{x}} \Vert \partial _t V \Vert _{L^2(0,T;H^{s_{\boldsymbol{x}}+1}(\varOmega ))}$. $\square$

##### Remark 16

Similarly to Remark 13, we observe that, due to the norm equivalence (10) in Remark 2, we readily have

$$\begin{aligned} \Vert U - U_{\boldsymbol{h}}\Vert _{L^2(Q_T)}+ \Vert V - V_{\boldsymbol{h}}\Vert _{L^2(Q_T)}\lesssim C(U,V) \left( h_{\boldsymbol{x}}^{s_{\boldsymbol{x}}+1}+h_t^{s_t+1}\right) . \end{aligned}$$

$$\blacksquare $$

### Numerical Results

In this section, we present two numerical tests in one space dimension to validate the stability and convergence of the proposed method.^1^ The first test involves a smooth solution. We demonstrate unconditional stability and observe convergence rates using standard space–time tensor product discretizations. In the second test, we consider a singular solution and still observe convergence, even though this case is not covered by our theory.

An important aspect not addressed in this work is the efficient implementation of the space–time method, including its potential reformulation as a time-marching scheme; we refer to [14, §5.1] for a detailed discussion.

#### Smooth Solution

As a first numerical test, we consider problem (1) in the domain $Q_T = \varOmega \times (0,1)$, with $\varOmega = (0,1)$, $c^2(x) = x+1$, $U_0(x) = \sin (\pi x)$, $V_0(x)=0$, and *F*(*x*, *t*) such that the exact solution is

52 $$\begin{aligned} U(x,t) = \sin ^2 \left( \frac{5}{4} \pi t \right) \sin (\pi x) + \sin (\pi x). \vspace{-0.2cm} \end{aligned}$$

**Fig. 1:**
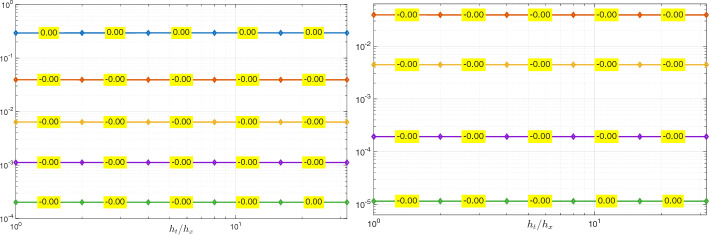
Test with the smooth solution given by (52). Relative errors in the norm $\mathcal {V}_{e,\boldsymbol{h}}(Q_T)$ for the discrete solution computed with the scheme in (25). The results are obtained by fixing the temporal mesh size $h_t$, decreasing the spatial mesh size $h_x$ and varying the polynomial degree *p*: , , , , . The left plot corresponds to maximal regularity splines ($p \ge 1$), while the right plot shows results obtained using $C^1$ splines ($p \ge 2$).

*Stability* We first demonstrate stability of the scheme in (25) with respect to the norm $\Vert \cdot \Vert _{\mathcal {V}_{e,\boldsymbol{h}}(Q_T)}$, as defined in (28), in line with the result of Corollary 1. The relative error in this norm is computed using first splines with maximal regularity and then $C^1$ splines in both time and space, all with the same polynomial degree *p* but different mesh sizes. Specifically, we fix the time step $h_t$ and progressively refine the spatial mesh size $h_x$. As shown in Figure 1, where the results for several values of *p* are reported, no oscillations in the error or instabilities are observed, and no CFL-type constraint of the form $h_t \le C h_x$ appears to be required. Here and below, we do not test with $C^0$ polynomials in time as, in this case, our scheme does not differ significantly from that in [18].

**Fig. 2:**
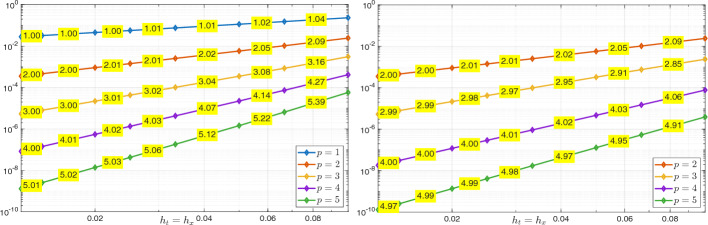
Test with the smooth solution given by (52). Relative errors in the norm $\mathcal {V}_{e,\boldsymbol{h}}(Q_T)$ for the discrete solution computed with the scheme in (25). The results are obtained by varying the spatial and temporal mesh sizes $h_t=h_x$ and varying the polynomial degree *p*. The left plot corresponds to maximal regularity splines, while the right plot shows results obtained using $C^1$-splines.

**Fig. 3:**
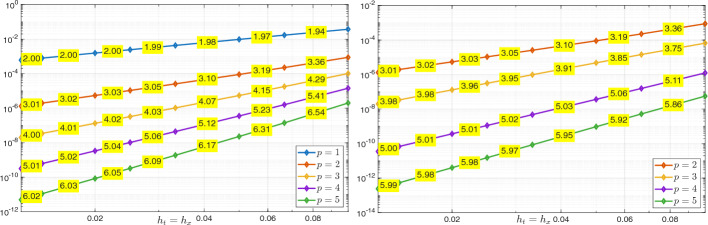
Test with the smooth solution given by (52). Relative errors in the norm $L^2(Q_T)$ for $U_{\boldsymbol{h}}$, the first component of the discrete solution computed with the scheme in (25). The results are obtained by varying the spatial and temporal mesh sizes $h_t=h_x$ and varying the polynomial degree *p*. The left plot corresponds to maximal regularity splines, while the right plot shows results obtained using $C^1$-splines.

*Convergence* To validate the convergence results established in Theorems 5 and 7, we compute the relative errors in the $\mathcal {V}_{e,\boldsymbol{h}}(Q_T)$ norm for $(U_{\boldsymbol{h}}, V_{\boldsymbol{h}})$, and in the $L^2(Q_T)$ norm for $U_{\boldsymbol{h}}$. When using splines in both space and time with equal polynomial degree *p* and uniform mesh size $h = h_t = h_x$, Theorem 5 predicts a convergence rate of order $\mathcal {O}(h^p)$ in the $\mathcal {V}_{e,\boldsymbol{h}}(Q_T)$ norm. Furthermore, from Theorem 7, we expect a convergence rate of order $\mathcal {O}(h^{p+1})$ in the $L^2$ norm. These theoretical rates are confirmed numerically in Figures 2 and 3 for both maximal regularity and $C^1$-splines. As expected, for $\Vert V-V_{\boldsymbol{h}}\Vert _{L^2(Q_T)}$, we obtained the same convergence rates as for $\Vert U-U_{\boldsymbol{h}}\Vert _{L^2(Q_T)}$. We do not report these results here, for brevity.

#### Singular Solution

As a second test, we consider the initial value boundary problem (1) with the non-smooth traveling wave solution of [7, §7, Problem 3]. The domain is given by $Q_T = \varOmega \times (0,1)$, with $\varOmega = (-1.5,1.5)$, and we set $F(x,t)=0$, $c(x)=1$. The initial conditions are chosen so that the exact solution is

53 $$\begin{aligned} U(x,t) = \omega (x-t+1) 1\!\!1_{>0}(x-t+1), \end{aligned}$$

where $1\!\!1_{>0}$ is the indicator function of $(0,\infty )$, and $\omega (s):= e^{-20(s-0.1)^2} - e^{-20(s+0.1)^2}$.

**Fig. 4:**
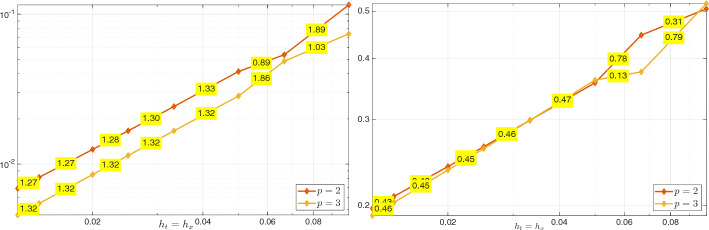
Test with the singular solution given by (53). Relative errors in the norm $L^2(Q_T)$ for $U_{\boldsymbol{h}}$ (left plot) and $V_{\boldsymbol{h}}$ (right plot), the two components of the discrete solution with the scheme in (25). Maximal regularity splines are employed in both space and time. The results are obtained by varying the spatial and temporal mesh sizes $h_t=h_x$, and for polynomial degrees $p=2,3$.

For $t \in (0,1)$, the solution satisfies $|U(\pm 1.5,t)| \le 10^{-17}$, so we impose homogeneous boundary conditions in practice. The solution *U*(*x*, *t*) is non-smooth along the line $x-~t+1=0$, due to a jump discontinuity in $\partial _t U$ across that line (since $\omega '(0)~=~8e^{-0.2} \ne 0$). Consequently, *U* lies in $H^{3/2-\varepsilon }(Q_T)$ for any $\varepsilon >0$, but not in $H^{3/2}(Q_T)$. Therefore, the velocity $V=\partial _t U$ fails to belong to $H^1(Q_T)$, so this example lies outside the theoretical framework of Theorem 5. We test the convergence of the discrete scheme (25) using uniform meshes and maximal regularity splines of degree *p* in both space and time. Although the regularity assumptions on $V=\partial _t U$ required by Theorem 5 are not satisfied, we observe numerical convergence rates in practice that match the expected ones: $\mathcal {O}(h^{3/2})$ for *U* and $\mathcal {O}(h^{1/2})$ for *V*. These results are presented in Figure 4.

**Fig. 5:**
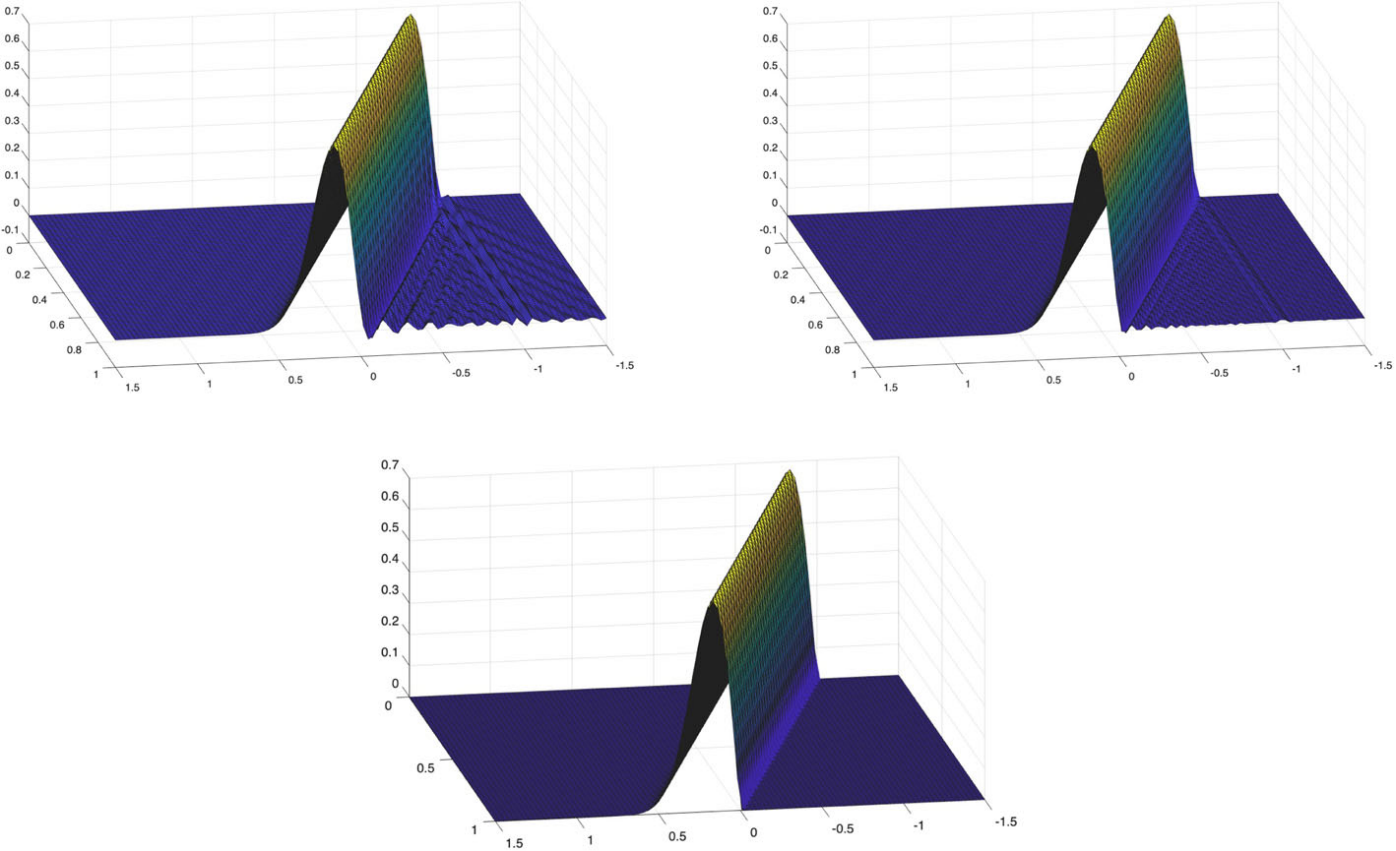
Test with the singular solution given in (53). The top plots show the discrete solutions computed with the scheme in (25), while the bottom panel displays the exact solution. Maximal regularity splines are used for the discretization in both space and time. Results are reported for $p_t=p_x=2$ with mesh sizes $h_x = h_t = 0.05$ (top left) and $h_x = h_t = 0.025$ (top right).

Furthermore, in Figure 5, we plot the space–time numerical solution $U_{\boldsymbol{h}}$ for two meshes with different size. We observe some numerical oscillations which, however, diminish as the mesh is refined. We obtained a similar behavior by increasing the polynomial degree. For approaches to prevent the appearance of such oscillations, we refer to discontinuous Galerkin methods in time [4, §7.2] or Galerkin least-squares stabilization techniques [22].

#### Mixed Boundary Conditions

In this example, we consider a problem with mixed Dirichlet and impedance boundary conditions in space. Although this configuration is not covered by our theoretical analysis, we show how our numerical scheme can be adapted to handle impedance boundary conditions and present a numerical experiment that shows optimal convergence rates also in this setting.

The test problem we consider reads as follows: find *U* such that

54 $$\begin{aligned} {\left\{ \begin{array}{ll} \partial _t^2 U(x,t) - \partial _x^2 U(x,t) = \frac{\pi ^2}{2} e^{- \frac{\pi }{2} t} \sin \left( \frac{\pi }{2} x\right) &  (x,t) \in (0,\frac{1}{2}) \times (0,1), \\ U(0,t) = 0 &  t \in (0,1], \\ \partial _x U(\frac{1}{2},t) + \partial _t U(\frac{1}{2},t) = 0 &  t \in (0,1], \\ U(x,0) = \sin \left( \frac{\pi }{2}x\right) &  x \in (0,\frac{1}{2}), \\ \partial _t U(x,0) = -\frac{\pi }{2} \sin \left( \frac{\pi }{2}x\right) &  x \in (0,\frac{1}{2}). \end{array}\right. } \end{aligned}$$

The analytical solution is given by $U(x,t) = e^{-\frac{\pi }{2} t} \sin \left( \frac{\pi }{2} x\right)$.

We utilize discrete spaces $S_{h_x}(0,\frac{1}{2}) \subset H^1_{0,\bullet }(0,\frac{1}{2})$ to enforce the Dirichlet condition at $x=0$. To incorporate the impedance boundary condition at $x=\frac{1}{2}$, we modify the bilinear form in (11) as follows:

$$\begin{aligned} \mathcal {A}^m((U_{\boldsymbol{h}},V_{\boldsymbol{h}}),(\lambda _{\boldsymbol{h}},\chi _{\boldsymbol{h}})) := \mathcal {A}((U_{\boldsymbol{h}},V_{\boldsymbol{h}}),(\lambda _{\boldsymbol{h}},\chi _{\boldsymbol{h}})) + (\partial _t U_{\boldsymbol{h}}(\tfrac{1}{2},\cdot ),\lambda _{\boldsymbol{h}}(\tfrac{1}{2},\cdot ))_{L_e^2(0,T)}. \end{aligned}$$

Alternatively, by exploiting the identity $\partial _t U = V$, one could define the modified bilinear form as

$$\begin{aligned} \widetilde{\mathcal {A}}^m((U_{\boldsymbol{h}},V_{\boldsymbol{h}}),(\lambda _{\boldsymbol{h}},\chi _{\boldsymbol{h}})) := \mathcal {A}((U_{\boldsymbol{h}},V_{\boldsymbol{h}}),(\lambda _{\boldsymbol{h}},\chi _{\boldsymbol{h}})) + (V_{\boldsymbol{h}}(\tfrac{1}{2},\cdot ),\lambda _{\boldsymbol{h}}(\tfrac{1}{2},\cdot ))_{L_e^2(0,T)}. \end{aligned}$$

**Fig. 6:**
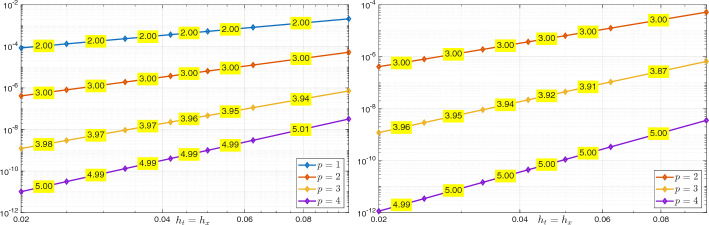
Test with the problem with mixed boundary condition in space given by (54). Relative errors in the $L^2(Q_T)$ norm for the discrete solution $U_{\boldsymbol{h}}$ computed with the scheme associated with the bilinear form $\mathcal {A}^m$. The results are obtained with polynomial degree $p=p_t=p_x$ and with $h_t=h_x$. The left plot corresponds to maximal regularity splines ($p \ge 1$), while the right plot shows results obtained using $C^1$ splines ($p \ge 2$).

Numerical experiments conducted with the two versions of the modified formulation yielded similar results. In Figure 6, we report the $L^2(Q_T)$ errors for $U_{\boldsymbol{h}}$ obtained using the formulation with $\mathcal {A}^m$. The corresponding convergence rates for $V_{\boldsymbol{h}}$ are identical and are therefore omitted.

#### $(2+1)$-Dimensional Test Case

We consider problem (1) in $Q_T = \varOmega \times (0,1)$, with $\varOmega = (0,1)^2$, $c(x_1,x_2) = 1$, $U_0(x_1,x_2) = 0$, $V_0(x_1,x_2)=0$, and $F(x_1,x_2,t)$ such that the exact solution is

55 $$\begin{aligned} U(x_1,x_2,t) = \sin (\pi x_1)\sin (\pi x_2) \sin ^2(t x_1 x_2). \end{aligned}$$

**Fig. 7:**
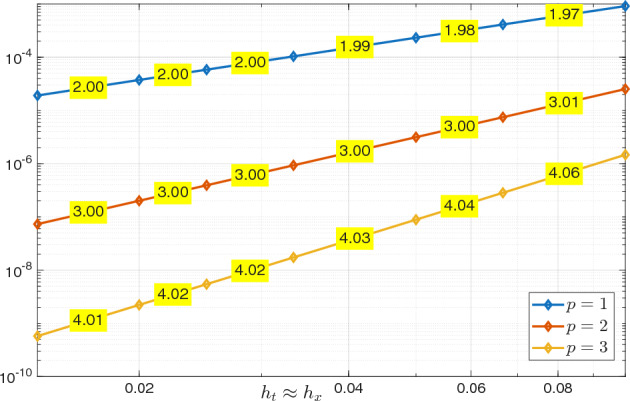
Test with the (2+1)-dimensional solution given by (55). Relative errors in the norm $L^2(Q_T)$ for $U_{\boldsymbol{h}}$ are shown. Maximal regularity splines are employed in time, and continuous finite elements on simplicial meshes are employed in space. The results are obtained by varying the spatial and temporal mesh sizes with $h_t \approx h_x$ and the polynomial degrees $p=p_t=p_{\boldsymbol{x}}.$

**Fig. 8:**
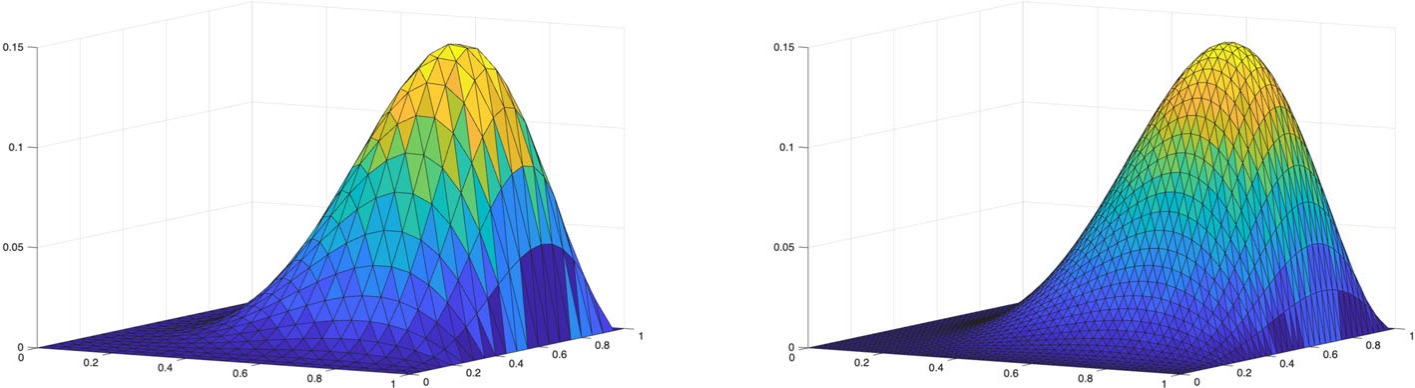
Test with the (2+1)-dimensional solution given in (55). The plots show the discrete solutions at $T=1$ computed with the scheme in (25). Maximal regularity splines are used in time and piecewise continuous polynomials on a simplicial mesh are used in space. Results are reported for $p_t=p_x=3$ with mesh sizes $h_{\boldsymbol{x}} \approx h_t = 0.05$ (left) and $h_{\boldsymbol{x}} \approx h_t = 0.025$ (right).

For the space discretization, we use continuous finite elements on structured, uniform simplicial meshes of the domain $\varOmega$. For the time discretization, we use maximal regularity splines on uniform meshes. We report in Figure 7 the relative errors in the $L^2(Q_T)$ norm for the wave field $U_{\boldsymbol{h}}$ obtained by employing polynomials of degree *p* for both space and time, meshes of size $h_t \approx h_x$. The theoretical rates are once again confirmed numerically in Figure 7. As expected, for $\Vert V-V_{\boldsymbol{h}}\Vert _{L^2(Q_T)}$, we obtained the same convergence rates as for $\Vert U-U_{\boldsymbol{h}}\Vert _{L^2(Q_T)}$. We do not report these results here, for brevity. Figure 8 presents the approximate solutions at the final time $T=1$ for two different levels of refinement.

**Fig. 9:**
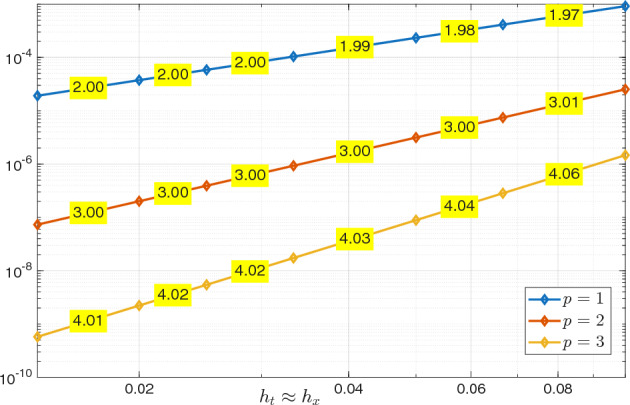
Same setting as in Figure 7, but using the numerical scheme obtained by removing the exponential weights in the $L^2(0,T)$ scalar products in the definition of the bilinear form $\mathcal {A}$ in (11).

As highlighted in Remark 8, although the exponential weights are necessary for the theoretical stability analysis, they do not appear to play a significant role from a numerical point of view. Figure 9 reports the same experiments as Figure 7, but with the bilinear form $\mathcal {A}$ in (11) computed without exponential weights. As can be observed, the resulting plots are identical.

## Conclusion

In this work, we introduced a conforming space–time tensor product discretization method for the wave equation based on a first-order-in-time variational formulation with exponential weights in time. The weights play a fundamental role in the stability analysis, allowing to prove unconditional well-posedness and optimal convergence rates with considerable flexibility in the choice of discrete spaces. However, from a practical point of view, they may be unnecessary. For instance, for the first-order-in-time formulation, they are proven not to be needed in the case of continuous piecewise polynomial approximations in time [18, 20], or when using maximal regularity splines in time [14]. Whether this is true in general remains an open question.

## Data Availability

The Matlab source code to reproduce this paper’s numerical experiments can be downloaded from https://github.com/MatteoFerrari11/XT-Waves-Exp-First-Order.git.

## Footnotes

1 All numerical tests are performed with Matlab R2024a. The codes used for the numerical tests are available in the GitHub repository [12].
